# Aging-Associated Changes in Cognition, Expression and Epigenetic Regulation of Chondroitin 6-Sulfotransferase *Chst3*

**DOI:** 10.3390/cells11132033

**Published:** 2022-06-27

**Authors:** David Baidoe-Ansah, Sadman Sakib, Shaobo Jia, Hadi Mirzapourdelavar, Luisa Strackeljan, Andre Fischer, Stepan Aleshin, Rahul Kaushik, Alexander Dityatev

**Affiliations:** 1 Molecular Neuroplasticity, German Center for Neurodegenerative Diseases (DZNE), 39120 Magdeburg, Germany; david.baidoe-ansah@dzne.de (D.B.-A.); jiashaobo3000@gmail.com (S.J.); hadi.mirzapourdelavar@dzne.de (H.M.); luisastrackeljan@aol.com (L.S.); alexander.dityatev@dzne.de (A.D.); 2 Department for Epigenetics and Systems Medicine in Neurodegenerative Diseases, German Center for Neurodegenerative Diseases, 37075 Goettingen, Germany; m.sadman.sakib@gmail.com (S.S.); a.fischer@eni-g.de (A.F.); 3 Clinic for Psychiatry and Psychotherapy, University Medical Center Goettingen (UMG), 37075 Goettingen, Germany; 4 Cluster of Excellence MBExC, University of Göttingen, 37075 Goettingen, Germany; 5 Center for Behavioral Brain Sciences (CBBS), 39106 Magdeburg, Germany; 6 Medical Faculty, Otto-von-Guericke University, 39120 Magdeburg, Germany

**Keywords:** aging, cognitive decline, extracellular matrix, carbohydrate sulfotransferase 3, 6-sulfation, epigenetic regulation

## Abstract

Understanding changes in the expression of genes involved in regulating various components of the neural extracellular matrix (ECM) during aging can provide an insight into aging-associated decline in synaptic and cognitive functions. Hence, in this study, we compared the expression levels of ECM-related genes in the hippocampus of young, aged and very aged mice. ECM gene expression was downregulated, despite the accumulation of ECM proteoglycans during aging. The most robustly downregulated gene was carbohydrate sulfotransferase 3 (*Chst3*), the enzyme responsible for the chondroitin 6-sulfation (C6S) of proteoglycans. Further analysis of epigenetic mechanisms revealed a decrease in H3K4me3, three methyl groups at the lysine 4 on the histone H3 proteins, associated with the promoter region of the *Chst3* gene, resulting in the downregulation of *Chst3* expression in non-neuronal cells. Cluster analysis revealed that the expression of lecticans—substrates of CHST3—is tightly co-regulated with this enzyme. These changes in ECM-related genes were accompanied by an age-confounded decline in cognitive performance. Despite the co-directional impairment in cognitive function and average *Chst3* expression in the studied age groups, at the individual level we found a negative correlation between mRNA levels of *Chst3* and cognitive performance within the very aged group. An analysis of correlations between the expression of ECM-related genes and cognitive performance in novel object versus novel location recognition tasks revealed an apparent trade-off in the positive gene effects in one task at the expense of another. Further analysis revealed that, despite the reduction in the *Chst3* mRNA, the expression of CHST3 protein is increased in glial cells but not in neurons, which, however, does not lead to changes in the absolute level of C6S and even results in the decrease in C6S in perineuronal, perisynaptic and periaxonal ECM relative to the elevated expression of its protein carrier versican.

## 1. Introduction

Plasticity in the nervous system underlies the ability of the brain to learn and modify existing behaviors and allows the organism to adapt to changing environments [1,2,3,4,5]. However, this ability decreases with age and highly correlates with the age-associated gradual accumulation of the brain ECM molecules at the protein level [6,7]. ECM in the brain exists in a condensed form known as perineuronal nets (PNN), mostly present around parvalbumin (PV)-expressing interneurons, but also as periaxonal “coats” and a more diffuse perisynaptic ECM around synapses on excitatory cells [8]. The expression of perineuronal and perisynaptic ECM is increased significantly with age in many brain areas, including the hippocampus and the cortex [7,9]. During postnatal development, the formation of perineuronal nets underlies the closure of the critical period which is marked by a significant reduction in the ability of the brain to structurally modify the already formed neuronal connections [6,10,11]. This helps to mature the brain circuitries and plays a critical role in brain function. Following this important developmental event, there is a life-long gradual accumulation of ECM proteoglycans in the brain that might underlie the age-dependent decline in cognitive functions such as learning and memory, which is a shared feature of both normal and pathological aging [12]. However, the molecular mechanism behind this age-dependent increase in ECM has been elusive so far.

**Figure 1:**
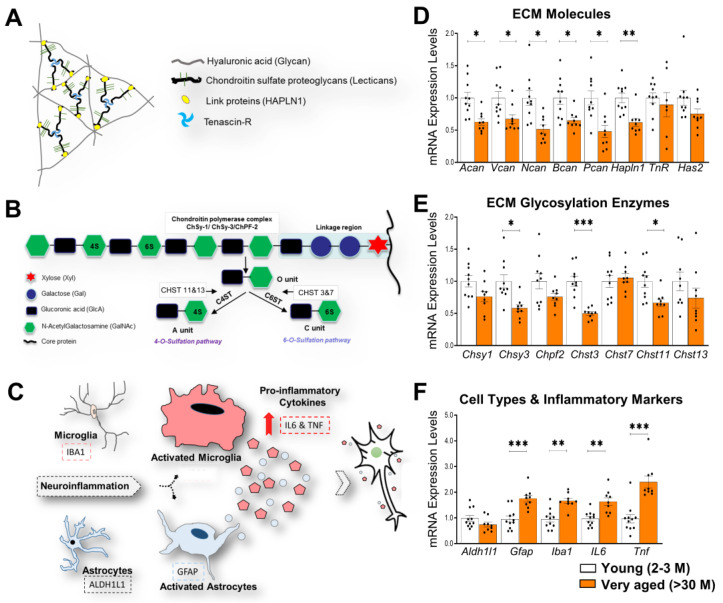
Downregulated ECM mRNA in the right hippocampus of >30 M-old mice. (**A**) Scheme depicting the basic principle of neural ECM organization. (**B**) GAG chains attached to core proteins of various types of CSPGs (e.g., aggrecan) are polymerized by the chondroitin polymerase complex which includes the chondroitin synthases 1 and 3 (Chsy1&3), and chondroitin polymerizing factor 2 (Chpf2). Unsulfated disaccharides (O unit) of GAG chains are further modified with sulfates yielding C4S (A unit) and C6S (C unit) by chondroitin 4-sulfotransferase (C4ST) and chondroitin 6-sulfotransferase (C6ST), respectively. (**C**) Neuroinflammatory microenvironment. (**D**) Genes encoding for core proteins of CSPGs; *Acan*, *Vcan*, *Ncan*, *Bcan*, and *Pcan*, were downregulated in >30 M-old mice compared to 2-32-3 M-old mice. (**E**) In >30 M-old mice, glycosylation enzymes were analyzed and C6 sulfotransferase *Chst3* was found to be downregulated compared to 2-3 M-old mice. (**F**) Inflammatory cytokines and markers for glial activation were upregulated in >30 M-old mice, a characteristic feature of aging, compared to 2-3 M-old mice. Bar graphs show mean ± SEM values. * *p* < 0.05, ** *p* < 0.01, and *** *p* < 0.001 represent significant differences between 2-3 M-old mice (*n* = 10) and >30 M-old mice (*n* = 9).

The backbone of neural ECM is a glycan, hyaluronic acid (HA), that is synthesized and anchored to the cell surface by hyaluronan synthases (HAS1-4) [13]. Other major constituents are chondroitin sulfate proteoglycans (CSPGs) of the lectican family, such as aggrecan (ACAN), brevican (BCAN), neurocan (NCAN), and versican (VCAN), as well as phosphacan (PCAN). CSPGs bind to HA via the amino-terminal hyaluronan binding domain, while the carboxy-terminal domain binds to the ECM glycoprotein tenascin-R (TNR), resulting in a net-like structure. Hyaluronan and proteoglycan link proteins (HAPLN1-4) stabilize the attachment of lecticans to HA [8] (Figure 1A). 

Lecticans are proteoglycans consisting of core proteins and a variable number of CS glycosaminoglycan (GAG) side chains that are long, unbranched polysaccharides made up of repeating disaccharide units of amino sugar, either N-acetylglucosamine (GlcNAc) or N-acetylgalactosamine (GalNAc), and glucuronic acid (GlcA). Chondroitin polymerizing factor 2 (CHPF2), chondroitin sulfate synthase 1 (CHSY1), and chondroitin sulfate synthase 3 (CHSY3) regulate the length of glycosaminoglycans (GAG) side chains that are added to the core proteins of CSPGs. GAG chains are further matured by other highly controlled modifications such as sulfation. Various carbohydrate sulfotransferase genes such as *Chst3*, *Chst7*, *Chst11*, and *Chst13* are important for sulfation of GAG chains mostly at position 4 (C4S) or 6 (C6S). CHST3 and CHST7 control the C6S, whereas CHST11 and CHST13 regulate the C4S of the GAG chains [14,15] (Figure 1B).

Studies show that the sulfation of GAGs is essential for both organ formation and function, especially with the C6S synthetized by the CHST3 enzyme [16,17]. Interestingly, C6S-containing CSPGs have been found to influence certain structural and physiological processes such as the differentiation of cardiac progenitors into cardiomyocytes during early development [18]. Findings from patients with skeletal abnormalities also showed additional cardiac abnormalities including valve regurgitations [19].

In humans, a genome-wide linkage study indicates that mutations in the *CHST3* gene result in structural defects such as skeletal dysplasia [20]. This has been observed in babies born with congenital bone malformations, including dislocated knee joints, dwarfism, and abnormal spine curvature [21]. The post-transcriptional regulation of the *CHST3* gene has been associated with the spinal condition lumbar disc degeneration (LDD) [22], which causes low back pain (LBP) in humans [23]. 

During CNS development, most CSPGs are 6-O-sulfated, which is essential for embryonic brain processes such as migration, differentiation, and the myelination of neurons [14]. The inhibitory role of the ECM in age-dependent decline in structural brain plasticity is thought to be related to the increasing ratio of C4S/C6S of the GAG chains attached to CSPGs. Four-sulfated GAG chains are highly inhibitory to axonal growth [24], whereas 6-O-sulfated GAG chains promote axonal growth [25]. Therefore, the C4S/C6S ratio appears to be a critical determinant of the functional involvement of GAG chains in neurite outgrowth, structural plasticity, and cognition, and this ratio is known to increase gradually not only at the end of the critical period but also throughout life [10,26,27]. However, the mechanisms behind the age-dependent increase in the C4S/C6S ratio are not yet clear.

Multiple studies have shown a gradual age-dependent physiological increase in neuroinflammation and the activation of astrocytes and microglia [28,29]. An involvement of these processes in cognitive decline was also shown [30]. In the CNS, neuroinflammation is marked by an increased expression of astrocytic and microglial markers, such as glial fibrillary acidic protein (GFAP) [31,32] and ionized calcium-binding adaptor molecule 1 (IBA1), respectively [33], along with the upregulation of proinflammatory cytokines such as interleukin 6 (IL-6) and tumor necrosis factor-alpha (TNFα) [29] (Figure 1C). Additionally, pathological conditions such as brain injury also lead to an increased activation of astrocytes that stimulate the secretion of several ECM molecules and ultimately results in the formation of the glial scar [31,34,35,36]. However, a possible increase in astrocyte activation during physiological aging and an increase in ECM production remains to be investigated. Thus, with the current study, we aimed to investigate the age-dependent alteration in the expression of ECM and related genes, including genes that regulate the expression of CS chains and their sulfation. Moreover, we correlated the expression of ECM-related genes with cognitive decline and neuroinflammation-related genes levels. We used qRT-PCR as a highly sensitive and reliable method to detect even minor systematic age-dependent differences in gene expression. Furthermore, we also investigated the potential underlying molecular mechanisms such as epigenetic changes that might be central to such gene expression regulation.

## 2. Materials and Methods

### 2.1. Animals

All animal experiments were conducted in accordance with the ethical animal research standards defined by German law and approved by the Ethical Committee on Animal Health and Care of the State of Saxony-Anhalt, Germany (license 42502-2-1343 DZNE). The present study used 68 male C57BL6/J mice, which included 34 young mice (2 to 3 months old, hereafter referred to as 2-3 M-old mice), 25 aged mice (22 to 24 months old, hereafter referred to as 22-24 M-old mice), and 9 very aged mice (>30 months old, hereafter referred to as >30 M-old mice). The numbers of mice used in each experiment are described in the text and figure legends. All mice were transferred to the research facility from the animal breeding facility and housed individually with food and water available ad libitum for at least 72 h before experiments under a reversed 12/12 light/dark cycle (light on 9 P.M.). All behavioral experiments were performed during the dark phase of the cycle, i.e., when mice are active, under constant temperature and humidity. 

### 2.2. Experimental Design

In the first batch of animals, we measured the cognitive decline in >30 M-old mice and 22-24 M-old mice compared to 2-3 M-old mice. All mice were sacrificed and brains were used for the gene expression analysis. To further investigate the changes in the expression of ECM genes at the earlier stages of aging, we conducted gene expression analysis on the second batch of twenty naive animals: ten 2-3 M-old mice and ten 22-24 M-old mice. Additionally, tissue samples from eight 2-3 M-old mice and eight 22-24-M-old mice were further used for FACS sorting and analysis of cell type-specific epigenetic changes of the selective genes.

### 2.3. Behavioral Analysis

All behavioral tests were conducted using ten 2-3 M-old mice and nine >30 M-old mice as well as six 2-3 M-old mice and eight 22-24 M-old mice. Anymaze 4.99 (Stoelting Co., Wood Dale, IL, USA) was used for capturing and analyzing animals’ performances during behavioral tests. Animals were subsequently tested using the open-field test, novel object location test (NOLT), and the novel object recognition test (NORT).

### 2.4. Open Field

Animals from each experimental group were put into an open-field arena (50 × 50 × 30 cm) [37,38] and allowed to freely move for 10 min while being recorded by an overhead camera. The arena was predefined into two parts, the central area (30 × 30 cm) and a peripheral area (the 10 cm area adjacent to the wall of the recording chamber). Total distance moved and time spent in the central/peripheral area were evaluated for animals’ general activity and anxiety.

### 2.5. Novel Object Location Test

The novel object location test was carried out in the same open-field arena. This test includes two phases: the encoding phase and the retrieval phase [39]. In the encoding phase, animals were allowed to explore the arena with a pair of identical objects for 10 min. Twenty-four hours later, in the retrieval phase, the animals were given 10 min to explore the arena again with the same objects, but one of them was placed in a novel position. Exploring time for an object located at familiar (F) and novel (N) positions and the discrimination ratio [(N − F)/(N + F)] × 100% were used to analyze animals’ behavior.

### 2.6. Novel Object Recognition Task

The novel object recognition test was also conducted in the open-field arena containing the encoding phase and retrieval phase as described previously [38,40]. Briefly, in the encoding phase, animals were allowed to explore the arena with a pair of identical objects. Twenty-four hours later, in the retrieval phase, one of the objects was replaced by a novel object that the animal had not seen before. Both the encoding and retrieval phases lasted 10 min. Exploration time in seconds for both objects was further analyzed by a trained observer who was blind to treatment conditions. Exploring time for familiar (F) and novel (N) objects and the discrimination ratio [(N − F)/(N + F)] × 100% were used to evaluate animals’ recognition memory.

### 2.7. Tissue Isolation

Animals were quickly decapitated and brain tissue was isolated in ice-cold PBS. Specific brain regions were further dissected and snap-frozen on dry ice and stored at −80 °C until further use or fixed. Hippocampal and cortical samples were isolated and processed. Left and right hippocampi from the second batch of animals were stored for only RNA extraction and downstream processing. For symmetry, we used the right hippocampus and right cortex for subsequent analysis.

### 2.8. RNA Extraction, cDNA Conversion, and QPCR

Total RNA was extracted from the frozen brain regions using EURx GeneMatrix DNA/RNA Extracol kit (Roboklon Cat. No. E3750) according to the manufacturer’s recommendations [41]. The RNA yield, purity, and integrity were determined with nano-drop and gel electrophoresis, respectively, to confirm the absence of genomic DNA. Furthermore, 1.5 µg of RNA was used for cDNA conversion using the High-Capacity cDNA Reverse Transcription Kit (Cat. 4368814). RT-qPCR was carried out using TaqMan gene expression array (Cat. 4331182) from Thermo Fisher Scientific (Waltham, MA, USA) using Quant-Studio-5 from Applied Biosystems. Nineteen genes were analyzed, comprising five CSPG genes, namely *Acan*, *Bcan*, *Ncan, Vcan*, and *Pcan*; two genes coding for link proteins, *Hapln1* and *TnR*; and eight genes regulating the synthesis of HA, GAGs and their sulfation levels, *Has2*, *Chpf2*, *Chsy1*, *Chsy3*, *Chst3*, *Chst7*, *Chst11*, and *Chst13*, respectively. Additionally, three cell type-specific markers, aldehyde dehydrogenase 1 family member L1 (*Aldh1l1*), glial fibrillary acidic protein (*Gfap*), ionized calcium-binding adaptor molecule 1 (*Iba1*), and two inflammatory markers, interleukin 6 (*IL6*) and tumor necrosis factor (*Tnf*), were also quantitatively measured and analyzed relative to expression of Glyceraldehyde 3-phosphate dehydrogenase (*Gapdh*) [42]. A list of Taqman probes is shown in Table 1. The mRNA levels of *Gapdh* were not significantly changed among 2-3 M-, 22-24 M-, and >30 M-old mice, making it a suitable candidate for the normalization of other genes. Additionally, due to the use of some cortical samples for the optimization of the RNA extraction protocol and insufficient cDNA for some of the genes, we could only run 5 samples per group for both *Bcan* and *Hapln1*. As such, we could not determine the mRNA levels of *Pcan* in 2-3 M-old mice and 22-24 M-old mice groups.

### 2.9. FACS Sorting for Neuronal and Glial Nuclei Extraction

Hippocampi from 2-3 M-old mice and 22-24 M-old mice from the third batch of animals were dissected, flash frozen and kept at −80 °C until further use. The nuclei extraction protocol was adapted from [43] with slight modifications. Briefly, left hippocampi were used for the extraction of nuclei to perform chromatin immunoprecipitation (ChIP). Frozen tissues were homogenized in low-sucrose buffer (LSB; 5 mM CaCl_2_-Applichem A3652,0500, 320 mM sucrose-Applichem A4737,5000, 0.1 mM EDTA-Invitrogen AM9260G, 5 mM MgAc_2_ -Sigma M5661-250G, 10 mM HEPES pH 8—Gibco 15630-056, 1 mM DTT—Roth 6908.2, 0.1% Triton X-100—Sigma T8787, 1× EDTA free Roche protease inhibitor cocktail—Sigma 5056489001) in 1.5 mL tubes. The crosslinking step was performed by adding 1% formaldehyde as the final concentration and was incubated for 10 min at room temperature. Furthermore, cross-linking was stopped by adding 125 mM glycine (Applichem A1067,0500) with incubation for 5 min at room temperature. Nuclei were spun down by centrifugation at 2000× *g* for 3 min at 4 °C. The remaining crude nuclear pellet was resuspended into LSB and homogenized with a mechanical homogenizer (IKA Ultraturax T10). The suspension was layered on 6 mL high-sucrose buffer (HSB; 3 mM MgAc_2_, 1 mM DTT, 1000 mM Sucrose, 10 mM HEPES pH 8, protease inhibitor) in oak-ridge tubes and centrifuged for 10 min at 3220× *g* at 4 °C. After removing the upper phase containing myelin debris, the resulting nuclear pellet was resuspended into the leftover buffer, transferred into microfuge tubes (Eppendorf DNA-low bind, 022431021), and centrifuged at 2000× *g* for 3 min to recover the nuclear pellet. Before staining, the nuclei pellet was resuspended into PBS containing Tween-20 and BSA (PBTB; 1% BSA, 0.2% Tween-20, EDTA-free protease inhibitor dissolved in 1× PBS) and Anti-NeuN-Alexa488-conjugated antibody (MAB377X) was added at 1:1000 dilution. After 1 h incubation at 4 °C, samples were washed twice with PBS and proceeded with the sorting in BD FACS Aria III. Sorted nuclei were collected into Falcon tubes, pelleted with brief centrifugation and pellets were flash frozen and saved at −80 °C until further processing for ChIP. In parallel, right hippocampi were used to perform nuclei extraction followed by RNA extraction using tissue from the same animals. A similar protocol was followed as described above, except the RNAse inhibitor (Promega N2615) which was used in all buffers and no crosslinking was conducted. After sorting, RNA was extracted using Trizol reagent (Sigma T9424) and RNA was kept at −80 °C until further processing.

### 2.10. Chromatin Immunoprecipitation and qPCR

Chromatin immunoprecipitation was performed as described previously [43] with slight modifications. Briefly, 0.2 µg chromatin was used for conducting H3K4me3 ChIP (1 µg antibody, ab8580). A 1:1 mixture of Protein A and G beads was used for pre-clearing and immunoprecipitation. ChIP DNA was subjected to qPCR using SYBR green reagents with primers targeting *Chst2* transcription start site ±200 bp. Primers were designed using primer BLAST (NCBI) by taking the genomic sequence and selecting for amplicon between 70 and 150 bp. The primer pair used for ChIP-qPCR was (5′-3′ direction) GGGCCTTTGTTCCCGACTTA (forward) and CTCGTCCTCAAGGGTAGGGA (reverse). The percent input method (% Input) was used to calculate H3K4me3 enrichment.

### 2.11. cDNA Preparation from Sorted Nuclear RNA and qPCR

RNA concentration from sorted nuclei was measured in Qubit 2.0 (Invitrogen) using RNA high-sensitivity kit. Three nanograms of RNA was used to prepare cDNA using SMART-Seq v4 Ultra Low Input Kit (Takara). Following cDNA preparation, equal amounts of cDNA were subjected to qPCR using primers (designed by Roche universal probe library tool) for *Chst3* mRNA. A standard curve was generated to calculate primer efficiency, which was used to obtain the normalized expression value (delta–delta Ct method), using DNA topoisomerase I (*Top1*) as a housekeeping gene [44]. Relative expression was calculated by normalizing to the 2-3 M samples. A list of probes used for sorted nuclei qPCR is shown in Table 2.

### 2.12. CHST3 Antibody Validation

Hippocampal cells from embryonic C57BL6/J mice (E18) were isolated and cultured as described earlier [45,46]. The cells were plated on polyethyleneimine-coated (Sigma-Aldrich; 408727-100 mL) 18 mm coverslips (Thermo Scientific) in 12-well plates with a cell density of 150,000 per well. Afterward, the cells were maintained in 1 ml of Neurobasal media (NB+ media) (Invitrogen) containing 2% B27 and 1% L-glutamine, and 1% Pen-Strep (Life Technologies). Cultured hippocampal cells were fed with 0.25 mL NB+ media on days in vitro (DIV) 14 and 17. On DIV 7, neurons and astrocytes were transduced with CHST3 overexpressing vectors (pAAV_GFAP_CHST3_BFP). On DIV 21, cells were fixed in 4% PFA and stained with CHST3 primary antibody to confirm the specificity of the antibody. Here, hippocampal neurons and astrocytes infected with CHST3 overexpressing vectors showed a stronger immunolabelling than the control culture (Figure S5). 

### 2.13. Immunohistochemistry

Young and aged mice were anesthetized with isoflurane, transcardially perfused with ice-cold phosphate-buffered saline (PBS), and fixed with 4% PFA. Mice brains were then extracted and immersed in 4% PFA overnight before being cryo-protected in 30% sucrose for 48 h. Finally, the brains were then chilled in 100% 2-methyl butane at −80 °C before sectioning into 50 μm-thick coronal sections. The sections were maintained in a floating solution (1 part ethylene glycol, 1 part glycerin, 2 parts PBS, pH = 7.2) at 4 °C. Using one section per animal for each staining, the sections were first washed 3 times in 120 mM phosphate buffer (PB) at a pH of 7.2 for 10 min, and then permeabilized with PB containing 0.5% Triton X-100 (Sigma, T9284) for 10 min at room temperature. Then, sections were blocked with a blocking solution (PB supplemented with 0.4% Triton X-100 and 10% normal goat serum, NGS (Gibco, 16210-064) for 1 h also at room temperature. Blocked sections were then incubated with primary antibodies (Suppl. Table S1) at 37 °C for 20 h, washed 3 times in PB for 10 min, followed by secondary antibodies (Suppl. Table S2) at room temperature for 3 h, and afterward also washed 3 times in PB for 10 min. Finally, sections were mounted on Superfrost glasses with Fluoromount (Sigma, F4680).

### 2.14. Data Acquisition, Processing, and Analysis

Overall, sections from six 2-3 M-old mice and seven 22-24 M-old mice were used for immunohistochemistry (IHC) analysis. Confocal images of stained sections were taken with the Zeiss LSM 700 microscope. For each staining, z-stacks of hippocampal CA1 images were obtained with the 63× objective at 0.5× digital zoom for CHST3 protein expression at perineuronal, periaxonal, and perisynaptic locations. The acquisition conditions were maintained throughout all imaging sessions to compare fluorescence intensity between samples. Images of CHST3 and C6S staining were processed and analyzed using a modified version of previously developed Fiji scripts [47]. Maximum projected images of CHST3 staining were analyzed by manually outlining neuronal cells using NeuN to measure neuronal CHST3 expression. For the glial expression of CHST3, we thresholded and outlined GFAP+ and IBA1+ cells. These markers are reliable in identifying astrocytes and microglia [38]. For perineuronal ECM measurements, the soma of PV+ cells was outlined manually, enlarged by 0.1µm, and a band of 0.6 µm was then created as the region of interest (ROI) to measure VCAN and C6S protein levels. A similar approach was used for periaxonal and perisynaptic measurements, except that axonal and synaptic selections were automatically made with Fiji macro. Here, thresholding was first used to select the axon initial segment (AIS) using the AnkG IHC, converted to a mask, and then outlined. These outlines were further filtered to remove non-AIS selections and a periaxonal band of 0.5 µm was created. For the perisynaptic ECM measurement, we first subtracted background in vGLUT1 images with the rolling ball value of 50 and Gaussian blur with an s-value of 1. Using the Find Maxima plugin in Fiji with a prominence of 2, size (0.3 to 1.0 μm^2^) and circularity (0.5–1.0), we identified and outlined synaptic puncta. Then, a perisynaptic band of 0.2 µm was created for each puncta. 

### 2.15. Statistical Analysis

Statistical analysis was performed using GraphPad Prism 7.0 (GraphPad Software Inc., La Jolla, CA, USA), Statistica 8.0 (StatSoft, St. Tulsa, OK, USA) and MATLAB 2019a (The Math Works, Inc., Portola Valley, CA, USA) software. All data are shown as mean ± SEM with *n* being the number of samples (mice). Asterisks in figures indicate statistical significance (with details in the figure legend or results). The hypothesis that experimental distributions follow the Gaussian law was verified using Kolmogorov–Smirnov, Shapiro–Wilk, or D’Agostinio tests. For pairwise comparisons, we performed Student’s *t*-test, where the samples qualified as normal; otherwise, the Mann–Whitney test was employed. Additionally, the paired *t*-test was used for paired comparisons or the Wilcoxon matched-pairs test if data did not pass the normality test. Holm–Sidak’s multiple comparisons *t*-test was used for independent comparisons. Spearman’s correlation (R) was used to calculate the distance between pairs of genes (1-R) when constructing clusterograms. Hierarchical clusters were linked using a “ward” metric. Significance and confidence intervals for multilevel regressions were calculated with bootstrapping (*n* resampling > 10^4^). The *p*-values represent the level of significance as indicated in figures by asterisks (* *p* < 0.05, ** *p* < 0.01, *** *p* < 0.001 and **** *p* < 0.0001), unless stated otherwise. 

To reveal the age-only correlational structure of gene expression patterns, we calculated a heatmap showing the pairwise correlations of genes after removing interactions within the age group (between-age correlations). To compensate for the effects of the within-group correlations, gene expression levels were randomly shuffled inside each group (*n* resampling > 10^4^). The average correlation value was used as a measure of “age-only” correlation for each gene pair. To determine the statistical significance, age values for each gene were randomized in the original data. For each of the resulting datasets (10^4^), the procedure for calculating age-only correlations described above was repeated. The final 10^4^ matrices were used as the H_0_ distribution to calculate the *p*-values. 

For the estimation of age-invariant gene pairwise correlations, we leveled out the effect of between-group correlations. To complete this task, gene expression levels were rank-normalized within each group independently, thus eliminating the effect of age on the average level of gene expression. Pairwise Spearman correlations were calculated for the resulting datasets. To determine the statistical significance, the approach described above (shuffling) was used.

To study the spatiotemporal dynamics of *CHST3* expression in the human brain, a published dataset was used [48]. To analyze the age-related dynamics of *Chst3* expression in various tissues and organs of mice, we used single-cell transcriptomics data obtained by Schaum and colleagues [49].

## 3. Results

To understand the interplay between cognitive decline, neuroinflammation, and high levels of ECM proteins in aging, we investigated the cognitive functions and mRNA expression of ECM molecules, as well as inflammatory markers in young and aged mice. We show, for the first time, that the reported reduction in 6-O-sulfated GAGs on the core proteins of CSPGs in aged mice comes from a rather complex pattern of age-dependent regulation of *Chst3* at multiple levels. 

### 3.1. Expression of CSPGs Core Proteins and Enzymes Regulating Synthesis and Sulfation of GAG Chains in the Hippocampus of >30 M-Old Mice and 22-24 M-Old Mice

Studies show that CSPG core proteins are highly upregulated in the aged brain [7,50]; therefore, we investigated if the upregulation of their mRNA transcripts might be the underlying mechanism of these high protein levels. Surprisingly, we observed significantly lower levels of mRNA of the core proteins of all CSPG genes, *Acan, Bcan, Ncan, Vcan*, and *Pcan* in >30 M-old mice (*p* = 0.0155, 0.0434, 0.0155, 0.0281, and 0.0155, respectively; Holm–Sidak’s multiple comparisons *t*-test). Furthermore, the link protein gene *Hapln1* was significantly reduced (*p* = 0.0081, Holm–Sidak’s multiple comparisons *t*-test). However, we did not observe any changes in the expression of the ECM glycoprotein *TnR* and the gene encoding for hyaluronan synthesis (*Has2*) (*p* = 0.5720 and 0.1799, respectively; Holm–Sidak’s multiple comparisons *t*-test) (Figure 1D). The *Gapdh* gene was used as a housekeeping gene to normalize the expression levels of these genes. To study the regulation of CSPG glycosylation levels, we measured the mRNA levels of genes encoding the glycosylating enzymes *Chsy1*, *Chsy3*, and *Chpf2*. We observed a significant reduction in the levels of *Chsy3* (*p* = 0.0150, Holm–Sidak’s multiple comparisons *t*-test). However, we did not detect any changes in other glycosylating genes such as *Chsy1* and *Chpf2* (*p* = 0.2384 and 0.2612, respectively; Holm–Sidak’s multiple comparisons *t*-test). In order to understand the sulfation levels of the glycoepitopes of GAGs, we investigated the expression levels of enzymes responsible for C6S (*Chst3* and *Chst7*) and C4S (*Chst11* and *Chst13*) sulfation. Interestingly, we revealed an approximately 50% reduction in the levels of *Chst3* (*p* = 0.0001) and 34% in *Chst11* (*p* = 0.0381) in >30 M-old mice (Figure 1E). In contrast, we did not see any changes in *Chst7* and *Chst13* mRNA levels (*p* = 0.6485 and 0.6225, respectively; Holm–Sidak’s multiple comparisons *t*-test). Collectively, these data suggest an involvement of some age-associated negative feedback mechanisms that might be involved in regulating not only the expression levels of CSPGs but also the glycosylating and sulfating enzymes. A strong age-associated increase in the activation of glial cells and neuroinflammatory cytokines has been shown previously [51]. Our gene expression data also support these observations, suggesting a contribution of transcriptional regulation to such upregulations. We detected a significant increase in activated astrocytic and microglial markers, *Gfap* (*p* = 0.0006) and *Iba1* (*p* = 0.0038), respectively, along with pro-inflammatory cytokines, *IL6* (*p* = 0.0016) and *Tnf* (*p* = 0.0001) (Figure 1F).

Our gene expression data from >30 M-old mice suggested that the high levels of ECM proteins might impart a strong negative feedback mechanism in the hippocampus that regulates the expression of ECM-related genes [52]. As these animals were at a very advanced stage of aging (>30 months), we hypothesized that the feedback mechanisms regulating the expression of ECM-related genes might not be active at the earlier stages of aging. Hence, we performed a gene expression analysis in 22-24 M-old mice. Interestingly, at this age, we observed no differences in the mRNA levels of core proteins of CSPGs in the hippocampus (Figure S1A). Moreover, a similar gene expression pattern was observed in the cortex (Figure S1B). In 22-24 M-old mice, we found a significant increase in the mRNA of glycosylation enzyme, *Chpf2* (*p* = 0.0373; Holm–Sidak’s multiple comparisons *t*-test), but no change in the expression of *Chsy1* and *Chsy3* (*p* = 0.4161 and 0.6776, respectively; Holm–Sidak’s multiple comparisons *t*-test). These changes were detected only in the hippocampus and not in the cortex (Figure S1C,D), suggesting some brain region-specific regulations. Remarkably, we consistently observed a strong downregulation of the mRNA levels of the *Chst3* gene in both hippocampus and cortex (*p* < 0.0001 and *p* = 0.0099, respectively; Holm–Sidak’s multiple comparisons *t*-test). Analysis of neuroinflammation/cell population markers revealed an increased expression of astrocytic activation marker *Gfap* in both the hippocampus and cortex of these mice (*p* = 0.0079 and *p* = 0.00013, respectively; Holm–Sidak’s multiple comparisons *t*-test). We also observed an increased expression of the microglial marker *Iba1* and the inflammatory marker *IL6*, but only in the hippocampus (*p* < 0.0001 and *p* = 0.0003, respectively; Holm–Sidak’s multiple comparisons *t*-test) and not in the cortex (*p* = 0.2997 and *p* = 0.2817, respectively; Holm–Sidak’s multiple comparisons *t*-test). These data suggest that aging-associated microgliosis and elevation in inflammatory markers, such as *IL6*, might happen in the hippocampus before it happens in the cortex (Figure S1E,F). However, another inflammatory cytokine gene, *Tnf*, was up-regulated in both the hippocampus and cortex (*p* < 0.0001 and *p* = 0.00013, respectively; Holm–Sidak’s multiple comparisons *t*-test; Figure S1E,F), probably due to the activation of astrocytes. These data suggest that the downregulation of *Chst3* might be one of the early ECM-related mechanisms that are affected by aging.

### 3.2. Relationships between the Expression of ECM-Related Genes in Three Studied Aged Groups

**Figure 2:**
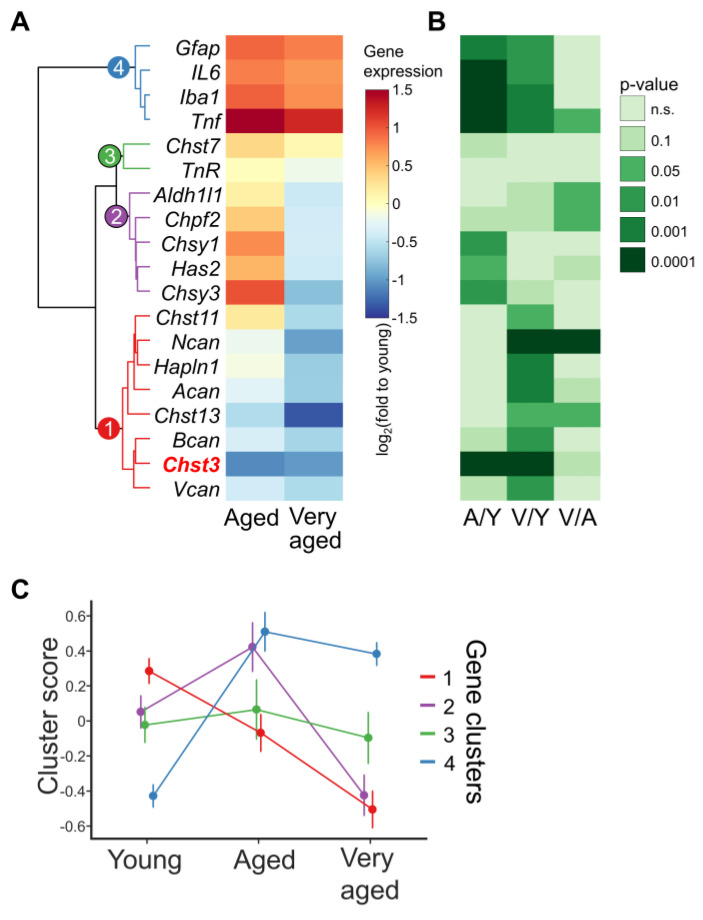
Comparison of aging-associated changes in expression of ECM, proinflammatory and cell-type-specific genes. (**A**) Changes in the expression of the studied genes compared to young animals (log_2_ fold change); (**B**) the level of statistical significance for comparisons aged vs. young (A/Y), very aged vs. young (V/Y) and very aged vs. aged (V/A) are shown on a heat map with different green intensity. These *p*-values were calculated by shuffling the mice-group relationship (*n* resampling 10^4^). (**C**) Four patterns of gene expression dynamics during aging. The studied genes were divided into clusters, using inter-age correlation as a measure of distance (see also Supplementary Figure S1). The details of the age-only correlation analysis are described in Materials and Methods.

Next, we aimed to investigate the global expression patterns of ECM-related genes in the hippocampus during aging. In the first step, genes were clustered according to their expression patterns in individual mice (Figure 2A,B). We distinguished four clusters. In the first cluster, we found PNN molecules (*Acan, Bcan, Vcan, Ncan*, and *Hapln1*) with the genes necessary for sulfation of GAGs, namely *Chst3, Chst13*, and *Chst11.* In the second cluster, we found the glycosylation enzymes (*Chsy1, Chsy3*, and *Chpf2*), clustered closely together with *Has2* and *Aldh1l1.* Surprisingly, the PNN molecule, *TnR*, and the sulfation enzyme, *Chst7*, showed no age-dependent or intra-age correlation with other genes, and we assigned them to a third cluster. The last cluster contained proinflammatory markers (*Gfap, Iba1, IL6*, and *Tnf*) (Figure 2A). To study the coordinated changes in gene expression within clusters, we calculated cluster scores. To do this, we performed principal component analysis (PCA) for each cluster separately and examined the effect of age on the values of the first principal component. We found a considerable age-dependent decline for cluster 1 (R = −0.7, *p*-value < 0.001; Figure 2C). The genes in the second cluster showed, on average, a bell-shaped dynamic of changes in mRNA levels, increasing in moderately aged animals and dropping significantly in very aged animals (Figure 2C). The third cluster showed no significant age-associated dynamics, whereas the proinflammatory cluster four showed a significant increase by 20 M, transitioning to a plateau in the >30 M group.

### 3.3. Cognitive Functions Are Impaired in >30 M and 22-24 M-Old Mice

**Figure 3:**
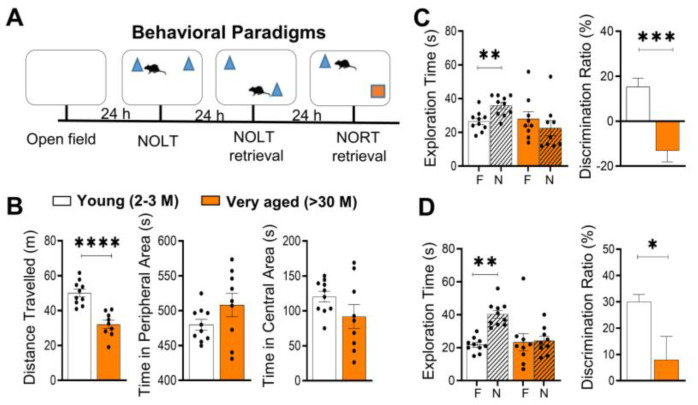
Impaired long-term memory in very aged mice (>30 M-old mice). Long-term memory was tested in >30 M-old mice and 2-3 M-old mice using the novel object location task (NOLT) and novel object recognition task (NORT). (**A**) Timeline for all cognitive tests. (**B**) >30 M-old mice traveled shorter distances compared to 2-3 M-old mice in the open field. Moreover, >30 M-old mice also spent equal time exploring familiar (F) and novel (N) locations and objects, indicating a failure to discriminate between objects in NOLT (**C**) and NORT (**D**). The difference in discrimination between >30 M-old mice and 2-3 M-old mice was further confirmed using discrimination ratio analysis. Bar graphs show mean ± SEM values for each animal. * *p* < 0.05, ** *p* < 0.01, *** *p* < 0.001 and **** *p* < 0.0001 represent significant differences between 2-3 M-old mice (*n* = 10) and >30 M-old mice (*n* = 9).

To identify the potential interaction between the aforementioned major age-dependent changes, we used NOLT and NORT tests to assess the cognitive status of nine >30 M-old mice and ten 2-3 M-old mice. Very old (>30 M) mice covered less distance compared to 2-3 M-old mice (traveling a mean distance of 32.1 ± 2.3 m vs. 50.6 ± 1.8 m, *p* < 0.001, unpaired Welch’s *t*-test; Figure 3B) in the open-field test. There was no difference in the time spent in the central area (92.0 ± 16.7 s vs. 124.7 ± 7.1 s; *p* = 0.1499, Mann–Whitney test; Figure 3B), suggesting no major changes in anxiety in >30 M-old mice. In NORT, >30 M-old mice seemed to have difficulty in discriminating between the familiar (F) and novel (N) objects with exploration time of 28.0 ± 4.262 s vs. 22.44 ± 4.476 s, respectively (*p* = 0.0508, Wilcoxon matched-pairs test). In contrast, 2-3 M-old mice showed strong discrimination between these objects (26.5 ± 1.833 s vs. 35.9 ± 1.906 s; *p* = 0.0098; Wilcoxon matched-pairs test). A comparison of discrimination ratio revealed a significant difference between the 2-3 M- and >30 M-old mice to distinguish novel object locations (15.22 ± 3.949 vs. −13.17 ± 4.965; *p* = 0.0003, Mann–Whitney test; Figure 3C). Similar to the NOLT, 2-3 M-old mice spent a significant amount of time exploring the novel objects with exploration times of 40.4 ± 2.1 s (N) and 21.3 ± 1.3 s (F) (*p* = 0.0020, Wilcoxon matched-pairs test) in the NORT test. However, >30 M-old mice spent a nearly equal time exploring both objects (24.2 ± 2.7 s (N) vs. 23.3 ± 5.3 s (F); *p* = 0.4805, Wilcoxon matched-pairs test). In line with this finding, the discrimination ratio analysis revealed a significant difference between >30 M-old and 2-3 M-old mice (30.0 ± 2.7 vs. 8.0 ± 8.9; *p* = 0.0182, Mann–Whitney test; Figure 3D). 

As we observed differences in expression of ECM-related genes between >30 M-old mice and 22-24 M-old mice, we then investigated the NOLT and NORT memory performance in 22-24 M-old mice. Here, 22-24 M-old mice covered less distance compared to 2-3 M-old mice (traveling a mean distance of 57.69 ± 2.9 m vs. 45.69 ± 3.1 m, *p* < 0.001, unpaired Welch’s *t*-test; Figure S2B) in the open-field task. There was no difference in time spent in the central area (121.1 ± 9.4 s vs. 125.1 ± 8.1 s; *p* = 0.8518, Mann–Whitney test; Figure S2B), indicating no major changes in anxiety in 22-24 M-old mice. However, we discovered a significant difference in the discrimination ratios between 2-3 M-old mice and 22-24 M-old mice for the NOLT test (36.14 ± 7.152 vs. −1.669 ± 8.872; *p* = 0.0127, Mann–Whitney test; Figure S2C). 

Contrary to the NORT findings in >30 M-old mice, we observed no difference between the NORT discrimination ratios for 2-3 M-old mice and 22-24 M-old mice (34.44 ± 6.285 vs. 26.62 ± 7.085; *p* = 0.5728, Mann–Whitney test; Figure S2D). In summary, we saw clear cognitive impairment in >30 M-old mice in both behavioral paradigms, but only NOLT memory impairment in 22-24 M-old mice compared to the 2-3 M-old mice.

### 3.4. Relationships between the Expression of ECM-Related Genes and Cognitive Performance

**Figure 4:**
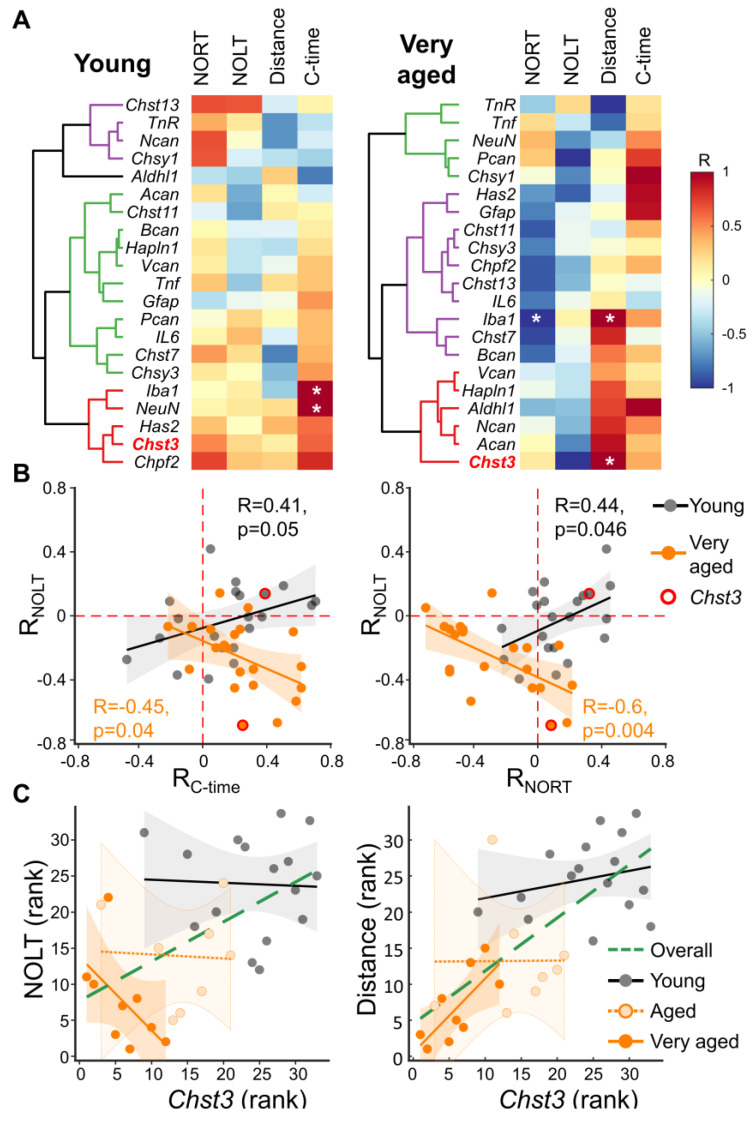
Spearman’s correlation between gene expression and behavioral characteristics of mice. (**A**) For each young and very aged animal, the readouts for the open-field test including the distance traveled and the time spent in the central zone of the arena (C-time) together with the performance of mice in NOLT and NORT tests were correlated with gene expression for each age subgroup. (**B**) Different gene/behavior bi-correlation patterns in different age cohorts were estimated. Herein, each point represents one gene in the coordinates of its correlation with the corresponding behavioral parameters: the left panel- NOLT and C-time; the right panel- NOLT and NORT. The average and confidence intervals of the linear regressions for the young (gray) and very aged (orange) groups are shown as lines and shaded areas, respectively. The correlation values for *Chst3* are highlighted with a red circle. (**C**) The correlation of *Chst3* mRNA expression with behavioral characteristics was measured. The correlations between *Chst3* mRNA levels and NOLT (left panel) or distance traveled (right panel) were calculated for young, old, and very aged individuals separately: grey, light, and deep orange, respectively. The overall correlation, confounded by age, is indicated by the green dashed line. Significant correlations for the left panel were: R = −0.68, *p* < 0.05 for very aged and R = 0.54, *p* < 0.001 for overall regressions. Significant correlations for the right panel were: R = 0.75, * *p* < 0.05 for very aged and R = 0.72, *p* < 0.001 for overall regressions.

Next, we performed a correlation analysis between the mRNA levels of different genes from 2-3 M and >30 M-old mice and their behavioral performance using the distance traveled, the time spent in the central area, and the discrimination ratios for cognitive tasks (NOLT and NORT). From 2-3 M-old mice, all genes were positively correlated with NORT, except for *Aldh1l1*, *Chst11*, and *Gfap*. However, almost half of the genes studied negatively correlated with NOLT, including *Chsy1*, *Aldh1l1*, *Acan*, *Chst11*, *Bcan*, *Hapln1*, *Vcan*, and *Tnf.* Regarding the relationship between the distance traveled and gene expression, we found the following genes to be negatively correlated to the distance: *Chst13*, *TnR*, *Ncan*, *Chsy1*, *Bcan*, *Hapln1*, *Vcan*, *IL6*, *Chst7*, *Chsy3*, and *Iba1*, whereas only *TnR*, *Ncan*, *Chsy1*, *Aldh1l1*, and *Acan* negatively correlated with the time spent in central area during the behavioral test (Figure 4A).

Next, we decided to study the high-level correlation of genes with various behavioral parameters. Figure 4B shows how the correlation of gene expression with NORT or time in the central area depends on the correlation of this gene with NOLT in different age groups. We found that the time spent in the central area positively correlated with the expression of the genes studied, whereas NORT showed a positive average correlation with the expression of the genes studied in young animals and a negative average correlation with the expression of the genes studied in very aged animals. The around-zero correlation of genes with NOLT in young mice was changed to an average negative correlation in aged experimental animals (Figure 4B). Regression analysis showed that in the 2-3 M age cohort, the correlation of genes with time spent in the central area and with NORT was positively related to their correlation with performance in NOLT. In other words, the correlation patterns of gene expression with NOLT, NORT, and central time are similar in young mice. At the same time, we observed a change in the sign of the regressions in very aged mice. This means that genes weakly correlated with NOLT show a strong correlation with the other behavioral parameters and vice versa (Figure 4B).

Then, using multilevel regression, we studied the correlation of *Chst3* with behavioral parameters in three ages, additionally performing behavioral testing and *Chst3* expression analysis in 22-24 M-old mice. We found a positive age-confounded (overall) correlation of *Chst3* with all the studied behavioral parameters (NOLT: R = 0.55, *p* = 0.0009; NORT: R = 0.37, *p* = 0.032; Distance: R = 0.74, *p* < 10^−5^; central time: R = 0.32, *p* = 0.07; Figure 4C). We also found that at the level of individual age cohorts, the correlations between *Chst3* and NOLT shifted from near-zero to negative values (R = −0.57, *p* = 0.043) in very aged mice. At the same time, the weakly positive correlations of *Chst3* expression and distance traveled were increased, reaching a maximum in the >30 M age cohort (R = 0.74, *p* = 0.022; Figure 4C). We also performed a multilevel correlation analysis of gene expression for each age cohort separately (Figure S3A). A subsequent co-clustering analysis showed that *Chst3* expression was closely related to CSPGs such as *Bcan*, *Acan*, and *Vcan*, but not to *Ncan* (Figure S3B). Another important observation is that *Chst7*, the enzyme responsible for C6 sulfation together with *Chst3*, showed weak co-clustering with *Chst3*.

We also performed a correlation analysis of gene expression at the group (age only) and within-group levels (age invariant). We found that the average expression of *Chst3* in each age group was strongly negatively correlated with proinflammatory genes. A similar negative correlation was observed for the CSPGs genes (Figure S4A). When we analyzed the correlation matrices at the group level, we also found a significant positive correlation between the CSPGs genes and *Chst3* (Figure S4A). 

This correlation may be caused either directly by the relationship between these genes or may be confounded by the general effect of aging. To discriminate between these options, we constructed correlation matrices after the deduction of the general effects of aging (Figure S4B). We found significant aging-independent positive correlations between *Chst3* and *Acan*, *Vcan* (*p* < 10^−4^), *Bcan* (*p* < 10^−3^), and a marginal correlation with *Ncan* (*p* = 0.047). These results are in good agreement with the clustering analysis performed above (Figure S3D).

### 3.5. Age-dependent Epigenetic Changes of the Chst3 Promoter in Non-Neuronal Cells

**Figure 5:**
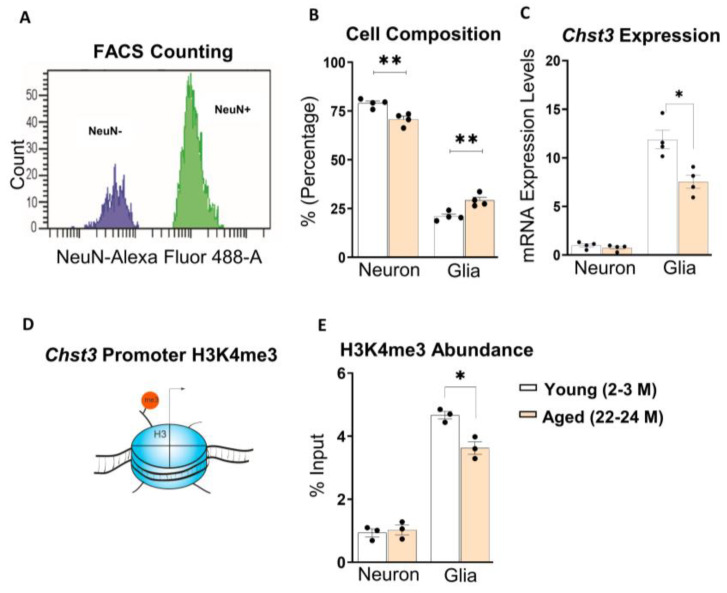
H3K4me3 is mostly enriched at the glial *Chst3* promoter in the hippocampus of 22-24 M-old mice. Changes in *Chst3* gene expression during aging were investigated specifically at the *Chst3* promoter in NeuN− positive and negative cells, which corresponds to neuronal and glial cells, respectively, in the hippocampus of 22-24 M-old mice and 2-3 M-old mice. (**A**) Representative neuronal and glial nuclei amounts were quantified and the cell composition for each study group was determined using FACS techniques with neuron-specific NeuN-antibody conjugated to Alexa Flour 488. (**B**) Thus, percentages of neurons and non-neuronal cells (predominantly glia) within test groups were compared. (**C**) No significant difference was observed in cell type-specific nuclear RNA-qPCR for the expression of C6 sulfotransferase *Chst3* in neurons, which was, however, downregulated in glia of 22-24 M-old mice. (**D**) Gene activation epigenetic mark, H3K4me3 at the *Chst3* promoter was investigated. (**E**) A statistical summary showing that using cell type-specific ChIP-qPCR, H3K4me3 abundance at *Chst3* promoter in neurons was found to be not different between 2-3 M-old mice and 22-24 M-old mice, whereas H3K4me3 was significantly less associated with *Chst3* promoter in glia of 22-24 M-old mice. Bar graphs show mean ± SEM values. * *p* < 0.05 and ** *p* < 0.01, represent significant differences between 2-3 M-old mice (*n* = 4 and 3 pairs) and 22-24 M-old mice (*n* = 4 and 3 pairs) for cell composition/*Chst3* expression and H3K4me3 abundance, respectively.

Next, we wanted to investigate the cell-type specific expression of the *Chst3*. Hippocampal tissues from 2-3 M-old mice and 22-24 M-old mice were sorted using the fluorescence-activated cell sorting (FACS) technique with NeuN antibody conjugated to Alexa-Flour 488-A (Figure 5A) to separate NeuN+ (neuronal) and NeuN− (non-neuronal) nuclei. We detected a higher proportion of NeuN+ cells than NeuN− in the hippocampus of the mice brain with significantly varying proportions, including 80% (NeuN+) vs. 20% (NeuN−) in 2-3 M-old mice compared to 70% (NeuN+, neurons) vs. 30% (NeuN−, predominantly glial cells) in 22-24 M-old mice (Figure 5B). Next, we performed RT-qPCR for the sorted cells to check the mRNA levels of *Chst3* expression. Interestingly, *Chst3* expression was more than 10-fold higher in glial cells when compared to neurons (Figure 5C). We detected a significant decrease in the mRNA levels of the *Chst3* gene in the NeuN− cell population but not in the NeuN+ cell population of 22-24 M-old mice (*p* = 0.0200 and *p* = 0.3126, respectively; Figure 5C). These data indicate that the observed decrease in the expression of *Chst3* upon aging most likely originates from the glial cell population in the 22-24 M-old mice hippocampus. 

To understand the mechanism behind the downregulation of *Chst3*, we studied the possible epigenetic modifications that might be responsible for these changes in *Chst3* expression during aging and are known to be linked to cognitive function [53,54,55,56]. Thus, we checked for changes in the activating H3K4me3 modification at the promoter region of the *Chst3* gene in both neuronal (NeuN+) and non-neuronal (NeuN−) cells. This is a very well-characterized epigenetic modification that has been shown to be centrally involved in regulating gene expression in eukaryotes [57,58]. 

Similar to the mRNA levels of *Chst3*, the levels of H3K4me3 at the promoter of *Chst3* gene in NeuN− glial sample were significantly higher in both 2-3 M-old mice and 22-24 M-old mice groups as compared to NeuN+ neuronal samples (Figure 5C,E). Moreover, we observed a significant reduction in the levels of H3K4me3 on the promoter of *Chst3* specifically in NeuN− cells but not in NeuN+ neurons of 22-24 M-old mice (*p* = 0.0220 and *p* = 0.6652, respectively; unpaired *t*-test with Welch’s correction; Figure 5E). These data highlight specific epigenetic changes at the promoter of *Chst3* in the NeuN− cell population that might underlie age-associated downregulation of *Chst3*. 

### 3.6. Age-Dependent Changes in the Expression and Function of CHST3 Protein

**Figure 6:**
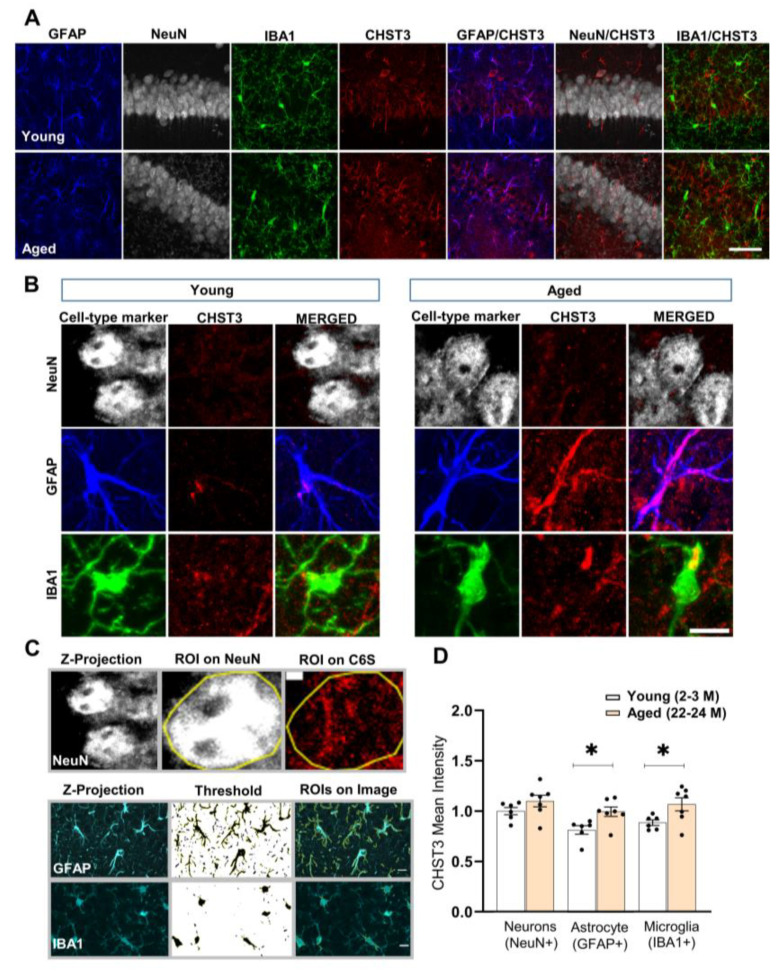
Cell-specific increase in CHST3 protein levels in the hippocampus of 22-24 M-old mice. (**A**) CHST3 protein levels were quantified in neurons, astrocytes, and microglia, which were identified using NeuN, GFAP, and IBA1 antibodies, respectively, in the hippocampus of 2-3 M-old mice and 22-24 M-old mice (scale bar, 50 µm). (**B**) Zoomed confocal images of CHST3 staining (scale bar, 10 µm). (**C**) Method of cell selecting. (**D**) The mean intensity of CHST3 was increased in astrocytes and microglia but not in neurons of 22-24 M-old mice compared to 2-3 M-old mice. Bar graph shows mean ± SEM values; * *p* < 0.05 represent significant difference between 2-3 M-old mice (*n* = 6) and 22-24 M-old mice (*n* = 7).

Next, we used IHC with antibodies against CHST3, and neuronal, astrocytic, and microglial markers to study age-dependent cell-type-specific changes in CHST3 protein levels in 22-24 M-old mice. We first validated the specificity of the CHST3 antibody in cultured mice hippocampal cells that were transduced to overexpress CHST3 proteins (pAAV_GFAP_CHST3_BFP) and showed increased immunolabeling (Figure S4). Surprisingly, using masks created from GFAP+ and IBA1+ cells (Figure 6C), we observed an increase in CHST3 protein levels in both astrocytes and microglia of 22-24 M-old mice compared to 2-3 M-old mice (*p* = 0.0422 for both astrocytes and microglia, respectively; Holm–Sidak’s multiple comparisons *t*-test). However, no difference was observed in NeuN+ cells between both groups of mice (Figure 6D). These results are contrary to the observed decreased mRNA levels of *Chst3* in NeuN− cells.

**Figure 7:**
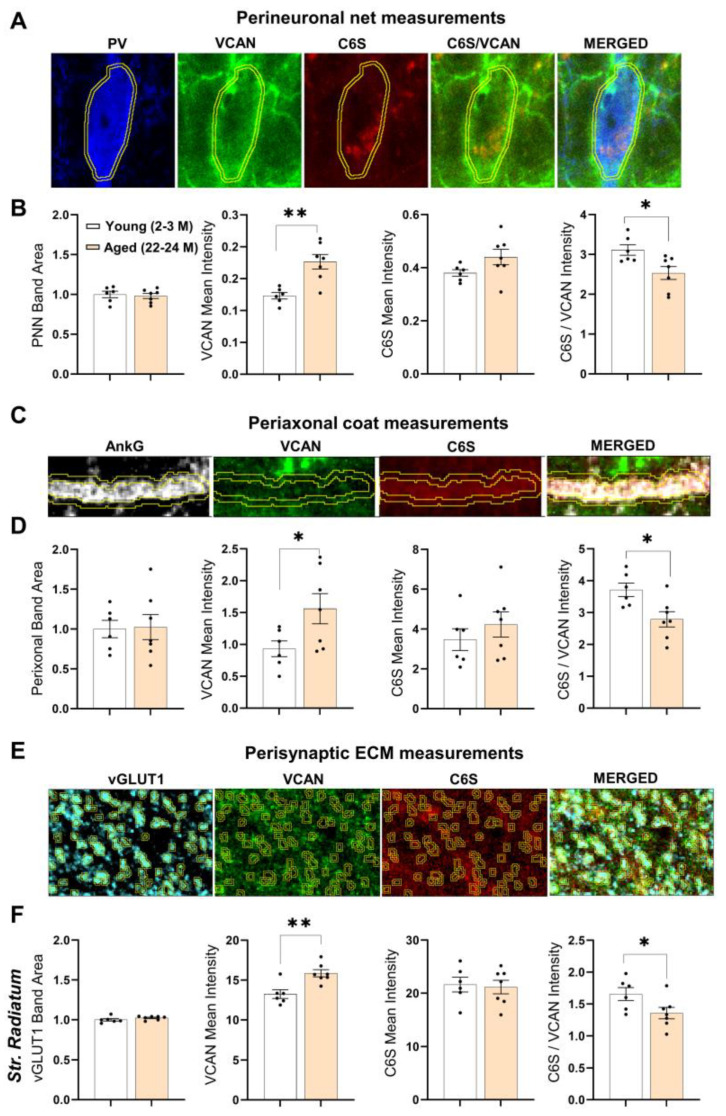
Reduced 6-sulfation of ECM in the hippocampus of 22-24 M-old mice. The functionality of CHST3 enzymes was estimated by using IHC with C6S and VCAN antibodies on hippocampal sections of 2-3 M-old mice and 22-24 M-old mice. Representative images of the method used to quantify the amount of VCAN core proteins associated with 6-sulfated GAGs around: (**A**) PV+ interneurons (perineuronal), (**C**) axon initial segment (periaxonal), and (**E**) excitatory synapses (perisynaptic). Computing a ratio between C6S and VCAN core protein expression levels showed a significant reduction in 6-sulfated ECM at the (**B**) perineuronal, (**D**) periaxonal, and (**F**) perisynaptic sites in 22-24 M-old mice relative to 2-3 M-old mice. Bar graphs show mean ± SEM values; * *p* < 0.05 and ** *p* < 0.01 represent significant differences between 2-3 M-old mice (*n* = 6) and 22-24 M-old mice (*n* = 7).

We then studied the CHST3 functionality in aged animals by immunolabeling of its chondroitin-6 sulfates and one of its substrates, the lectican VCAN. VCAN appeared as the best choice as a recent study indicates VCAN is the major carrier of 6-O-sulfates [59]. We measured the perineuronal, periaxonal, and perisynaptic VCAN and C6S content in a 0.6 μm proximity around PV+ interneurons (Figure 7A,C,E). From perineuronal measurements, we observed a significant increase in VCAN core proteins levels in 22-24 M-old mice (*p* = 0.0024; unpaired *t*-test with Welch’s correction; Figure 7B) and no detectable changes in C6S. However, normalizing the C6S fluorescent intensity to that of VCAN, we found a reduction in the C6S/VCAN ratio in PNNs of 22-24 M-old mice relative to 2-3 M-old mice (*p* = 0.0192; unpaired *t*-test with Welch’s correction; Figure 7B). Similar findings were obtained for the periaxonal and perisynaptic (especially in *stratum radiatum*) expression of C6S. We observed an increase in VCAN core protein levels around the axon initial segment (AIS), whereas the C6S/VCAN ratio was decreased (*p* = 0.0431 and *p* = 0.0149, respectively; unpaired *t*-test with Welch’s correction; Figure 7D). Furthermore, from the perisynaptic ECM measurements in the *stratum radiatum*, we observed increased VCAN core protein levels around excitatory synapses and a reduction in the C6S/VCAN ratio (*p* = 0.0042 and *p* = 0.0490, respectively; unpaired *t*-test with Welch’s correction; Figure 7F). 

### 3.7. Cross-Species and Cross-Tissue Analysis of Chst3 Expression

A recent functional study demonstrates the benefits of forced *Chst3* overexpression for the novel object recognition in aged mice [60]. This data and our gene expression analysis suggest that a treatment enhancing *Chst3* expression can be of interest for cognitive enhancement in aging. To compare our findings in mice with the regulation of *CHST3* in the human brain during aging, we used a published transcriptomic dataset [48] (GSE25219). We revealed that the age-related dynamics of *CHST3* expression in the human brain vary considerably in different brain regions, and, moreover, may have different trends in pre- and postnatal periods of development (Figure S6). The decline in expression of hippocampal *Chst3* that we observed in mice was in line with the negative trend of *CHST3* expression in the human hippocampus, amygdala and cerebellar cortex during aging. However, in the superior temporal and medial prefrontal cortex there was no aging-associated change in *CHST3* in humans, while in the most of studied areas, including the primary auditory (A1), motor (M1), sensorimotor (S1) and visual (V1) cortex, there was an increase in *CHST3* levels with aging (Figure S6).

We also compared our mouse tissue data for the three age groups with data from a study of changes in the transcriptomes of different mouse organs during development [49]. These data show complex nonlinear dynamics of *Chst3* expression levels during mouse brain development. Nevertheless, these transcriptomic data are consistent with our findings and also demonstrate a negative trend in the brain during aging after 25 months (Figure S7). An even more prominent decline in the expression of *Chst3* was found in the aging heart and skin. A reduction in *Chst3* expression was observed after 20 months in limb muscles, bone, marrow, and kidney tissues. However, in the spleen, white blood cells, small intestine and mesenteric fat there was a strong upregulation of *Chst3* expression in aged mice (Figure S7), clearly showing a tissue-specific pattern of aging-associated *Chst3* changes.

## 4. Discussion

The expression of CSPG core proteins and GAG chains shapes the embryonic, postnatal, and adult stages of brain development [8,61], by influencing cell migration, differentiation, and maturation [62]. Additionally, recent evidence suggests that aging increases the protein content of CSPGs in the brain and this correlates with a number of age-dependent structural and physiological changes [7]. These changes, including the reduction in synaptogenesis, synaptic transmission, and plasticity in various brain regions such as the hippocampus and prefrontal cortex, are associated with cognitive impairment [63]. It is noteworthy that little or no information exists regarding the mechanisms behind age-dependent changes in CSPG protein content, as this could be through the parallel increase in transcription and translation of CSPG genes or the disruption of the regulatory mechanisms. Therefore, in this study, we aimed to decipher transcriptional changes in the expression of ECM-related genes that might underlie age-dependent changes in CSPG protein content followed by protein analysis of identified targets using IHC.

In a proteomics study, Vegh and colleagues showed a time-dependent increase in the expression of some ECM molecules such as HAPLN1, BCAN, and NCAN. They also performed a more detailed immunoblotting analysis to confirm the upregulation of these molecules in the hippocampus, as a function of age. Using WFA labeling, they showed an age-dependent increase in the more specialized form of ECM, that is, PNNs [7]. These findings were also observed in other models of brain pathologies associated with aging such as depression [50]. However, these studies did not elucidate the mechanism underlying the increased levels of PNN proteins in aged mice. According to our findings, the reported high protein levels of PNN components are not caused by an increase in the transcription of the CSPG core proteins. In fact, the expression of CSPGs is decreasing with aging. 

Furthermore, we found a significant increase in the gene expression levels of enzymes, such as *Chpf2*, responsible for adding the GAG side chains to CSPG core proteins in the 22-24 M-old animals. However, we could not observe this increase in >30 M-old animals; rather, there was a significant decrease in the expression of *Chsy3*. This suggests that at the initial stages of aging, the ECM molecules might be secreted with longer GAG chains. However, in the later stages of aging, there is not only decreased expression of ECM core proteins but they also potentially carry shorter GAG chains. This further suggests the potential involvement of feedback mechanisms activated by the accumulation of ECM in the regulation of both core proteins and the length of GAG chains associated with them. These conclusions are merely based on the expression levels of these enzymes and we cannot rule out the additional level of regulation due to changes in the protein and activity levels of these enzymes. In addition, the lack of differences between aged and young mice in terms of the expression of glycosylation enzymes suggests that the high WFA intensity in aged animals as reported by Vegh and colleagues may not be due to an increase in GAG synthesis. This is because WFA binds to the terminal N-acetylgalactosamine residues of the GAG chains of CSPGs [64,65,66], and its intensity reflects the amount and maturity of PNNs [67]. Accordingly, the high WFA intensity reported might be a result of the accumulation of CSPGs and GAGs, possibly due to the reduced degradation of CSPGs. 

From this study, we can conclude that the expression of *Chst3* within all three age groups strongly relates to the expression of *Bcan, Vcan*, and *Acan*. The *Chst3* gene produces the CHST3 enzyme, which catalyzes the sulfation at the sixth position of carbon in chondroitin sulfates, which has been found to be low in previous studies [68]. Collectively, our data suggest that as aging progresses the secreted CSPGs not only have fewer 6-O-sulfated GAG chains but also shorter GAGs due to lower expression of GAG-adding enzyme complex of *Chsy3*, *Chsy1*, and *Chpf2* [69,70]. We further demonstrate that the *Chst3* gene seems to be highly downregulated not only in the hippocampus and cortex of 22-24 M mice but also in the hippocampus of >30 M mice, suggesting that the decreased expression of C6 sulfating enzymes such as *Chst3* might be an important global mechanism behind the increase in C4S/C6S. Interestingly, we observed an increase in the expression of *Chst13*, a carbohydrate sulfotransferase that is responsible for the C4-sulfation of GAGs in the cortex but not the hippocampus of 22–24 M-old mice. The increased ratio of C4S/C6S on the GAGs that are present on PNN forming core protein aggrecan attracted the most attention as a potential mechanism behind the structural plasticity-restraining function of ECM. Moreover, the increase in the ratio is largely attributed to the decrease in C6S GAGs [27,71]. This suggests a brain area-specific regulation and provides an additional mechanism for the increase in the ratio of C4S/C6S in the cortex. In line with our findings, studies using transgenic mice overexpressing CHST3 observed a reduction in the C4S/C6S ratio. This resulted in an impairment of PNN formation and reduced maturation of PV interneurons, including a reduction in their inhibitory effects [68]. Additionally, CHST3 overexpression resulted in persistent cortical plasticity [10]. On the contrary, the downregulation of *Chst3* during early development leads to the closure of the critical period and suppression of developmental plasticity. 

Here, we investigated the epigenetic changes in the regulation of the *Chst3* gene in a cell type-specific manner. We found that *Chst3* mRNA levels are significantly downregulated, specifically in NeuN− cells that mostly represent the glial cell population, but not in neurons. These changes are accompanied by the decreased abundance of H3K4me3 on the promoter region of *Chst3*, explaining the potential decrease in the mRNA levels of the gene. Therefore, from these data, we suspect that the age-dependent increased ratio of C4S/C6S might be due to not only an increase in the expression of enzymes carrying out C4S but also to a decrease in the expression or functionality of enzymes responsible for the synthesis of C6S. As *Chst3* plays a major role in the process of 6-sulfation, we expected to see an age-dependent decrease in *Chst3* expression, especially in non-neuronal cells of the brain. To confirm these objectives, we used IHC to examine how *Chst3* translates and functions at the protein level.

Surprisingly, contrary to mRNA levels, the expression of CHST3 protein was found to be upregulated in glia but not in neurons within the hippocampus of 22-24 M-old mice. This work, to our knowledge, is the first to quantify the cell-specific expression of CHST3 in the hippocampus of aged mice. The higher content of CHST3 could mean that: 1. the increased post-transcriptional modification and translation of mRNA species and/or 2. decreased degradation of these proteins. For example, m6A RNA-methylation was shown to control mRNA translation and thereby decouple gene-expression levels from protein production [72]. Both hypo- and hyper-methylation are observed in neurodegenerative diseases [73]. It would therefore be interesting to study *Chst3* mRNA methylation levels in glial cells.

The specificity of CHTS3 antibodies was validated in our study, as hippocampal neurons and astrocytes cultures infected with AAV to overexpress CHST3 showed increased immunolabeling as compared to controls (Figure S5). Since CHST3 levels are elevated in the aged hippocampus, it raises questions about its functional outcome, as previous studies have shown that there is an age-dependent reduction in 6-sulfated GAGs as well as increased PNN content [7,14]. As a recent study indicates VCAN as the major carrier of 6-sulfated GAGs [59], in addition to the consistent correlation between *Chst3* and *Vcan* expression levels observed in this study, we used VCAN as the CHST3 substrate to estimate the functionality of CHST3. By staining hippocampal slices for VCAN and C6S, we discovered a high amount of VCAN core proteins in aged mice and a lower ratio of 6-sulfated GAGs relative to *Vcan* expression levels. Our finding is in line with previous studies that examined the ratio of 6-sulfated to 4-sulfated GAGs and showed this ratio decreases with aging [14,17]. Moreover, it has already been established that the onset of 4-sulfation of GAGs coincides with PNN formation as 4-sulfated GAGs are more resistant to proteolysis [74]. A strong correlation between ECM proteolysis and expression of 6-sulfated CSGPS has previously been reported [68]. A reduction in C6S/VCAN or C6S/C4S indicates that CSPGs in aged mice are less susceptible to proteolysis. Thus, the reduced proteolysis of CSPGs might result in the reported higher levels of CSPG proteins.

Both neurons and astrocytes secrete ECM molecules into the extracellular space and contribute to the integrity of ECM in the brain [75,76]. Several studies have reported that the increased activation of astrocytes leads to the secretion of large amounts of ECM molecules after brain injury [31], which ultimately results in the formation of the glial scar [77]. However, in the aging brain, though there is increased activation of astrocytes, as indicated by elevated levels of *Gfap* expression [78], the decrease in the mRNA expression of neural ECM molecules strongly goes against the notion that an age-dependent increase in astrocytic activation might lead to an increase in the production of ECM-related genes. A gradual increase in brain inflammation is the hallmark of the aging brain as reported previously in 24-month-old mice [78,79]. In accordance with previous studies, we found a significant and consistent upregulation of inflammatory genes such as *Gfap*, *Iba1*, *Il6*, and *Tnf* during aging. The increased expression of *Gfap* suggests an increased activation of astrocytes. Noteworthy, we did not observe any changes in the expression of another astrocytic marker, *Aldh1l1*, which is a pan-astrocyte marker, hence making it a good candidate to relate to the total number of astrocytes, which is independent of the activation state of the astrocytes [80,81]. These data suggest that the number of astrocytes does not change dramatically, and the increased activation of astrocytes is likely not to induce the elevated synthesis of the studied ECM molecules.

From the clustering analysis, we found an interesting age-dependent effect on the expression of the majority of PNN molecules together with some sulfation enzymes, especially with *Chst3*. This makes sense as the expression and sulfation of GAGs depend on the expression levels of CSPG core proteins. In the next step, to dissect the mechanisms by which the upregulation of ECM affects cognitive performance in aging, we investigated the cognitive decline in aged mice and correlated their performance with the expression levels of various ECM-related genes. Strikingly, the multilevel regression analysis showed a strong correlation of *Chst3* expression with some behavioral parameters like object discrimination in NOLT, as a function of age. Additionally, there has been strong evidence linking memory impairments in aged mice to *Chst3* expression [60]; the deletion of *Chst3* resulted in memory loss as early as 11 weeks in mice. A comparison of coefficients of correlations between ECM gene expression and performance of mice in NORT versus NOLT revealed that, in young mice, genes positively correlating with performance in NORT also positively correlated with performance in NOLT, but in aged mice, there was an obvious trade-off in positive effects of multiple ECM-related genes on one form of learning at expense of another. 

Additionally, a complex pattern of aging-associated changes in *CHST3* gene expression in different human brain regions as well as in different mouse tissues may represent a trade-off in optimization of multiple brain and body functions. Hence, drugs increasing the *Chst3* expression and improving novel object recognition, as one could expect from the study of Yang and colleagues (2021), may have unwanted cognitive and other side effects. For instance, upregulated *Chst3* is found in human glioma tissues, where it may enhance cell viability, migration, and invasion of glioma cells [82]. The downregulation of *Chst3* may be beneficial to diminishing the accumulation of excessive macrophages in some tissue [83] and hence drugs increasing *Chst3* expression might have pro-inflammatory effects. Thus, a more comprehensive analysis of conditions when and how to target *Chst3* is necessary to explore its beneficial cognitive effects.

## 5. Conclusions

In this study, we show a decreased expression of *Chst3* mRNA specifically in the non-neuronal (glial) cell types along with a decrease in the gene expression-promoting epigenetic marker in the hippocampus of aged mice. Although the CHST3 protein levels were increased in contrast to their mRNA levels, the fraction/degree of CSPG 6-sulfation was still reduced in PNNs, perisynaptically and around AIS. This indicates that the age-dependent changes in CSPG 6-sulfation are a net result of the reduced expression of *Chst3* mRNA and enhanced synthesis/reduced degradation of CHST3 protein, which, however, does not lead to detectable changes in the absolute level of C6S and even result in C6S reduction relative to the expression level of its major substrate, VCAN. As the proteolysis of CSPGs is known to depend on the sulfation of GAGs, with 6-sulfated species being susceptible to degradation, the observed reduction in C6S might also reflect an age-dependent dysregulation in the expression and activity of ECM-degrading enzymes, so less ECM proteoglycans are degraded and hence accumulate in the extracellular space, but those which are still degraded are preferentially 6-sulfated.

## Figures and Tables

**Table 1:** Taqman probes used for real-time PCR analysis.

Gene	Full Description	Dye	Reference Sequence
*Acan*	Mouse_Aggrecan_Mm00545794_m1	FAM	NM_007424.2
*Vcan*	Mouse_Versican_Mm01283063_m1	FAM	NM_001081249.1
*Ncan*	Mouse_Neurocan_Mm00484007_m1	FAM	NM_007789.3
*Bcan*	Mouse_Brevican_Mm00476090_m1	FAM	NM_001109758.1
*Pcan*	Mouse_PTPRZ1_Phosphacan_Mm00478484_m1	FAM	NM_001081306.1
*Hapln1*	Mouse_HAPLN1_Mm00488952_m1	FAM	NM_013500.4
*TnR*	Mouse_TenascinR_Mm00659075_m1	FAM	NM_022312.3
*Has2*	Mouse_Hyaluronan Synthase 2 _Mm00515089_m1	FAM	NM_008216.3
*Chsy1*	Mouse_Chondroitin sulfate synthase 1_ Mm01319178_m1	FAM	NM_001081163.1
*Chsy3*	Mouse_ Chondroitin sulfate synthase 3_ Mm01545329_m1	FAM	NM_001081328.1
*Chpf2*	Mouse_Chondroitin polymerizing factor 2_Mm00659795_m1	FAM	NM_133913.2
*Chst3*	Mouse_Chst3_Mm00489736_m1	FAM	NM_016803.3
*Chst7*	Mouse_Chst7_Mm00491466_m1	FAM	NM_021715.1
*Chst11*	Mouse_Chst11_Mm00517562_m1	FAM	NM_021439.2
*Chst13*	Mouse_Chst13_Mm01186255_s1	FAM	NM_027928.1
*Aldh1l1*	Aldehyde Dehydrogenase 1 Family, Member L1_Mm03048957_m1	FAM	NM_027406.1
*Gfap*	Glial Fibrillary Acidic Protein _Mm01253033_m1	FAM	NM_001131020.1
*Iba1*	Allograft Inflammatory Factor 1_Iba1_Mm00479862_g1	FAM	NM_019467.2
*Il6*	Interleukin 6_IL6_Mm00446190_m1	FAM	NM_031168.1
*Tnf*	TNF-Alpha_Mm00443258_m1	FAM	NM_001278601.1
*Gapdh*	Mouse_GAPDH_Mm99999915_g1	FAM	NM_001289726.1

**Table 2:** Probes used for sorted nuclei qPCR.

Gene	Species	Reference Sequence		Sequence (5′ to 3′)
*Chst3*	*Mus musculus*	NM_007424.2	Fw	CCACAGCAGCCAGATCTTC
			Rv	TGGGGGACACTCTGATCCT
*Top1*	*Mus musculus*	NM_009408.2	Fw	TGCCTCCATCACACTACAGC
			Rv	CGCTGGTACATTCTCATCAGG

## Data Availability

Data supporting reported results can be obtained upon request to the corresponding authors (A.D. and S.A.).

## Supplementary Materials

The following supporting information can be downloaded at: https://www.mdpi.com/article/10.3390/cells11132033/s1: Table S1. Primary antibodies and their dilutions; Table S2. Secondary antibodies and their dilutions. Figure S1. Downregulation of C6 sulfotransferase *Chst3* in the hippocampus and cortex of 22-24 M-old mice; Figure S2. Impaired long-term NOLT memory in aged mice (22-24 M-old mice); Figure S3. Age-specific correlations in gene expression; Figure S4. Within and between age gene expression correlation patterns; Figure S5. Validation of CHST3 antibody specificity; Figure S6. Comparison of age-related dynamics of *CHST3* expression in different regions of the human brain; Figure S7. Comparison of age-related dynamics of *Chst3* expression in different mice tissues.

## References

[1] Burke S.N., Barnes C.A. (2006). Neural plasticity in the ageing brain. Nat. Rev. Neurosci..

[2] Sengpiel F. (2007). The critical period. Curr. Biol..

[3] Galván A. (2010). Neural plasticity of development and learning. Hum. Brain Mapp..

[4] Attardo A., Lu J., Kawashima T., Okuno H., Fitzgerald J.E., Bito H., Schnitzer M.J. (2018). Long-Term Consolidation of Ensemble Neural Plasticity Patterns in Hippocampal Area CA1. Cell Rep..

[5] Ribic A., Crair M.C., Biederer T. (2019). Synapse-Selective Control of Cortical Maturation and Plasticity by Parvalbumin-Autonomous Action of SynCAM 1. Cell Rep..

[6] Dityatev A., Fellin T. (2008). Extracellular matrix in plasticity and epileptogenesis. Neuron Glia Biol..

[7] Vegh M.J., Rausell A., Loos M., Heldring C.M., Jurkowski W., van Nierop P., Paliukhovich I., Li K.W., del Sol A., Smit A.B. (2014). Hippocampal extracellular matrix levels and stochasticity in synaptic protein expression increase with age and are associated with age-dependent cognitive decline. Mol. Cell. Proteom..

[8] Dityatev A., Schachner M. (2003). Extracellular matrix molecules and synaptic plasticity. Nat. Rev. Neurosci..

[9] Dityatev A., Brückner G., Dityateva G., Grosche J., Kleene R., Schachner M. (2007). Activity-dependent formation and functions of chondroitin sulfate-rich extracellular matrix of perineuronal nets. Dev. Neurobiol..

[10] Miyata S., Komatsu Y., Yoshimura Y., Taya C., Kitagawa H. (2012). Persistent cortical plasticity by upregulation of chondroitin 6-sulfation. Nat. Neurosci..

[11] Carulli D., Kwok J.C., Pizzorusso T. (2016). Perineuronal Nets and CNS Plasticity and Repair. Neural Plast..

[12] Richard A.D., Lu X.H. (2019). “Teaching old dogs new tricks”: Targeting neural extracellular matrix for normal and pathological aging-related cognitive decline. Neural Regen. Res..

[13] Weigel P.H. (2015). Hyaluronan Synthase: The Mechanism of Initiation at the Reducing End and a Pendulum Model for Polysaccharide Translocation to the Cell Exterior. Int. J. Cell Biol..

[14] Mikami T., Kitagawa H. (2013). Biosynthesis and function of chondroitin sulfate. Biochim. Et Biophys. Acta (BBA)—Gen. Subj..

[15] Miyata S., Kitagawa H. (2017). Formation and remodeling of the brain extracellular matrix in neural plasticity: Roles of chondroitin sulfate and hyaluronan. Biochim. Biophys. Acta Gen. Subj..

[16] Sugahara K., Mikami T., Uyama T., Mizuguchi S., Nomura K., Kitagawa H. (2003). Recent advances in the structural biology of chondroitin sulfate and dermatan sulfate. Curr. Opin. Struct. Biol..

[17] Uyama T., Ishida M., Izumikawa T., Trybala E., Tufaro F., Bergström T., Sugahara K., Kitagawa H. (2006). Chondroitin 4-O-Sulfotransferase-1 Regulates E Disaccharide Expression of Chondroitin Sulfate Required for Herpes Simplex Virus Infectivity. J. Biol. Chem..

[18] Prinz R.D., Willis C.M., van Kuppevelt T.H., Klüppel M. (2014). Biphasic Role of Chondroitin Sulfate in Cardiac Differentiation of Embryonic Stem Cells through Inhibition of Wnt/β-Catenin Signaling. PLoS ONE.

[19] Thiele H., Sakano M., Kitagawa H., Sugahara K., Rajab A., Höhne W., Ritter H., Leschik G., Nürnberg P., Mundlos S. (2004). Loss of chondroitin 6-O-sulfotransferase-1 function results in severe human chondrodysplasia with progressive spinal involvement. Proc. Natl. Acad. Sci. USA.

[20] Srivastava P., Pandey H., Agarwal D., Mandal K., Phadke S.R. (2017). Spondyloepiphyseal dysplasia Omani type: CHST3 mutation spectrum and phenotypes in three Indian families. Am. J. Med. Genet. Part A.

[21] Hermanns P., Unger S., Rossi A., Perez-Aytes A., Cortina H., Bonafé L., Boccone L., Setzu V., Dutoit M., Sangiorgi L. (2008). Congenital Joint Dislocations Caused by Carbohydrate Sulfotransferase 3 Deficiency in Recessive Larsen Syndrome and Humero-Spinal Dysostosis. Am. J. Hum. Genet..

[22] Song Y.-Q., Karasugi T., Cheung K.M.C., Chiba K., Ho D.W.H., Miyake A., Kao P.Y.P., Sze K.L., Yee A., Takahashi A. (2013). Lumbar disc degeneration is linked to a carbohydrate sulfotransferase 3 variant. J. Clin. Investig..

[23] Deyo R.A., Weinstein J.N. (2001). Low back pain. N. Engl. J. Med..

[24] Smith-Thomas L.C., Stevens J., Fok-Seang J., Faissner A., Rogers J.H., Fawcett J.W. (1995). Increased axon regeneration in astrocytes grown in the presence of proteoglycan synthesis inhibitors. J. Cell Sci..

[25] Wang H., Katagiri Y., McCann T.E., Unsworth E., Goldsmith P., Yu Z.-X., Tan F., Santiago L., Mills E.M., Wang Y. (2008). Chondroitin-4-sulfation negatively regulates axonal guidance and growth. J. Cell Sci..

[26] Carulli D., Pizzorusso T., Kwok J.C.F., Putignano E., Poli A., Forostyak S., Andrews M.R., Deepa S.S., Glant T.T., Fawcett J.W. (2010). Animals lacking link protein have attenuated perineuronal nets and persistent plasticity. Brain.

[27] Foscarin S., Raha-Chowdhury R., Fawcett J.W., Kwok J.C.F. (2017). Brain ageing changes proteoglycan sulfation, rendering perineuronal nets more inhibitory. Aging.

[28] Godbout J.P., Chen J., Abraham J., Richwine A.F., Berg B.M., Kelley K.W., Johnson R.W. (2005). Exaggerated neuroinflammation and sickness behavior in aged mice following activation of the peripheral innate immune system. FASEB J..

[29] Lynch M.A. (2010). Age-related neuroinflammatory changes negatively impact on neuronal function. Front. Aging Neurosci..

[30] Pluvinage J.V., Haney M.S., Smith B.A.H., Sun J., Iram T., Bonanno L., Li L., Lee D.P., Morgens D.W., Yang A.C. (2019). CD22 blockade restores homeostatic microglial phagocytosis in ageing brains. Nature.

[31] George N., Geller H.M. (2018). Extracellular matrix and traumatic brain injury. J. Neurosci. Res..

[32] Lyons A., Lynch A.M., Downer E.J., Hanley R., O’Sullivan J.B., Smith A., Lynch M.A. (2009). Fractalkine-induced activation of the phosphatidylinositol-3 kinase pathway attentuates microglial activation in vivo and in vitro. J. Neurochem..

[33] Ito D., Tanaka K., Suzuki S., Dembo T., Fukuuchi Y. (2001). Enhanced expression of Iba1, ionized calcium-binding adapter molecule 1, after transient focal cerebral ischemia in rat brain. Stroke.

[34] Beggah A.T., Dours-Zimmermann M.T., Barras F.M., Brosius A., Zimmermann D.R., Zurn A.D. (2005). Lesion-induced differential expression and cell association of Neurocan, Brevican, Versican V1 and V2 in the mouse dorsal root entry zone. Neuroscience.

[35] Cregg J.M., DePaul M.A., Filous A.R., Lang B.T., Tran A., Silver J. (2014). Functional regeneration beyond the glial scar. Exp. Neurol..

[36] Fawcett J.W., Asher R.A. (1999). The glial scar and central nervous system repair. Brain Res. Bull..

[37] Holter S.M., Einicke J., Sperling B., Zimprich A., Garrett L., Fuchs H., Gailus-Durner V., Hrabe de Angelis M., Wurst W. (2015). Tests for Anxiety-Related Behavior in Mice. Curr. Protoc. Mouse Biol..

[38] Kaushik R., Morkovin E., Schneeberg J., Confettura A.D., Kreutz M.R., Senkov O., Dityatev A. (2018). Traditional Japanese Herbal Medicine Yokukansan Targets Distinct but Overlapping Mechanisms in Aged Mice and in the 5xFAD Mouse Model of Alzheimer’s Disease. Front. Aging Neurosci..

[39] Vogel C., Marcotte E.M. (2012). Insights into the regulation of protein abundance from proteomic and transcriptomic analyses. Nat. Rev. Genet..

[40] Antunes M., Biala G. (2012). The novel object recognition memory: Neurobiology, test procedure, and its modifications. Cogn. Process..

[41] Ventura Ferreira M.S., Bienert M., Müller K., Rath B., Goecke T., Opländer C., Braunschweig T., Mela P., Brümmendorf T.H., Beier F. (2018). Comprehensive characterization of chorionic villi-derived mesenchymal stromal cells from human placenta. Front. Aging Neurosci..

[42] Meldgaard M., Fenger C., Lambertsen K.L., Pedersen M.D., Ladeby R., Finsen B. (2006). Validation of two reference genes for mRNA level studies of murine disease models in neurobiology. J. Neurosci. Methods.

[43] Halder R., Hennion M., Vidal R.O., Shomroni O., Rahman R.U., Rajput A., Centeno T.P., van Bebber F., Capece V., Garcia Vizcaino J.C. (2016). DNA methylation changes in plasticity genes accompany the formation and maintenance of memory. Nat. Neurosci..

[44] Penna I., Vella S., Gigoni A., Russo C., Cancedda R., Pagano A. (2011). Selection of candidate housekeeping genes for normalization in human postmortem brain samples. Int. J. Mol. Sci..

[45] Dityatev A., Dityateva G., Sytnyk V., Delling M., Toni N., Nikonenko I., Muller D., Schachner M. (2004). Polysialylated neural cell adhesion molecule promotes remodeling and formation of hippocampal synapses. J. Neurosci..

[46] Minge D., Senkov O., Kaushik R., Herde M.K., Tikhobrazova O., Wulff A.B., Mironov A., van Kuppevelt T.H., Oosterhof A., Kochlamazashvili G. (2017). Heparan Sulfates Support Pyramidal Cell Excitability, Synaptic Plasticity, and Context Discrimination. Cereb. Cortex..

[47] Strackeljan L., Baczynska E., Cangalaya C., Baidoe-Ansah D., Wlodarczyk J., Kaushik R., Dityatev A. (2021). Microglia Depletion-Induced Remodeling of Extracellular Matrix and Excitatory Synapses in the Hippocampus of Adult Mice. Cells.

[48] Kang H.J., Kawasawa Y.I., Cheng F., Zhu Y., Xu X., Li M., Sousa A.M.M., Pletikos M., Meyer K.A., Sedmak G. (2011). Spatiotemporal transcriptome of the human brain. Nature.

[49] Tabula Muris Consortium, Overall Coordination, Logistical Coordination, Organ Collection and Processing, Library Preparation and Sequencing, Computational Data Analysis, Cell Type Annotation, Writing Group, Supplemental Text Writing Group (2018). Principal investigators: Single-cell transcriptomics of 20 mouse organs creates a Tabula Muris. Nature.

[50] Koskinen M.-K., van Mourik Y., Smit A.B., Riga D., Spijker S. (2020). From stress to depression: Development of extracellular matrix-dependent cognitive impairment following social stress. Sci Rep..

[51] Sochocka M., Diniz B.S., Leszek J. (2017). Inflammatory Response in the CNS: Friend or Foe?. Mol. Neurobiol..

[52] Bonnans C., Chou J., Werb Z. (2014). Remodelling the extracellular matrix in development and disease. Nat. Rev. Mol. Cell Biol..

[53] Cruz C., Della Rosa M., Krueger C., Gao Q., Horkai D., King M., Field L., Houseley J. (2018). Tri-methylation of histone H3 lysine 4 facilitates gene expression in ageing cells. Elife.

[54] Kerimoglu C., Sakib M.S., Jain G., Benito E., Burkhardt S., Capece V., Kaurani L., Halder R., Agís-Balboa R.C., Stilling R. (2017). KMT2A and KMT2B Mediate Memory Function by Affecting Distinct Genomic Regions. Cell Rep..

[55] Mei Q., Xu C., Gogol M., Tang J., Chen W., Yu X., Workman J.L., Li S. (2019). Set1-catalyzed H3K4 trimethylation antagonizes the HIR/Asf1/Rtt106 repressor complex to promote histone gene expression and chronological life span. Nucleic Acids Res..

[56] Pu X., Xiao Q., Kiechl S., Chan K., Ng F.L., Gor S., Poston R.N., Fang C., Patel A., Senver E.C. (2013). ADAMTS7 cleavage and vascular smooth muscle cell migration is affected by a coronary-artery-disease-associated variant. Am. J. Hum. Genet..

[57] Howe F.S., Fischl H., Murray S.C., Mellor J. (2017). Is H3K4me3 instructive for transcription activation?. Bioessays.

[58] Santos-Rosa H., Schneider R., Bannister A.J., Sherriff J., Bernstein B.E., Emre N.C., Schreiber S.L., Mellor J., Kouzarides T. (2002). Active genes are tri-methylated at K4 of histone H3. Nature.

[59] Chelini G., Durning P., O’Donovan S., Klengel T., Balasco L., Berciu C., Boyer-Boiteau A., Bozzi Y., McCullumsmith R., Ressler K.J. (2021). Proteoglycan Clusters as a Site of Coordinated, Multi-Dendritic Plasticity. https://www.biorxiv.org/content/10.1101/2021.10.04.462691v1.

[60] Yang S., Gigout S., Molinaro A., Naito-Matsui Y., Hilton S., Foscarin S., Nieuwenhuis B., Tan C.L., Verhaagen J., Pizzorusso T. (2021). Chondroitin 6-sulphate is required for neuroplasticity and memory in ageing. Mol. Psychiatry.

[61] Williams M.E., de Wit J., Ghosh A. (2010). Molecular Mechanisms of Synaptic Specificity in Developing Neural Circuits. Neuron.

[62] Urbán N., Guillemot F. (2014). Neurogenesis in the embryonic and adult brain: Same regulators, different roles. Front. Cell Neurosci..

[63] Mencio C.P., Hussein R.K., Yu P., Geller H.M. (2021). The Role of Chondroitin Sulfate Proteoglycans in Nervous System Development. J. Histochem. Cytochem..

[64] Young N.M., Williams R.E. (1985). Assignment of lectins specific for D-galactose or N-acetyl-D-galactosamine to two groups, based on their circular dichroism. Can. J. Biochem. Cell Biol..

[65] Ajmo J.M., Eakin A.K., Hamel M.G., Gottschall P.E. (2008). Discordant localization of WFA reactivity and brevican/ADAMTS-derived fragment in rodent brain. BMC Neurosci..

[66] Miyata S., Nadanaka S., Igarashi M., Kitagawa H. (2018). Structural Variation of Chondroitin Sulfate Chains Contributes to the Molecular Heterogeneity of Perineuronal Nets. Front. Integr. Neurosci..

[67] Slaker M.L., Harkness J.H., Sorg B.A. (2016). A standardized and automated method of perineuronal net analysis using Wisteria floribunda agglutinin staining intensity. IBRO Rep..

[68] Miyata S., Kitagawa H. (2016). Chondroitin 6-Sulfation Regulates Perineuronal Net Formation by Controlling the Stability of Aggrecan. Neural Plast..

[69] Bowman K.G., Bertozzi C.R. (1999). Carbohydrate sulfotransferases: Mediators of extracellular communication. Chem. Biol..

[70] Li X., Tu L., Murphy P.G., Kadono T., Steeber D.A., Tedder T.F. (2001). CHST1 and CHST2 sulfotransferase expression by vascular endothelial cells regulates shear-resistant leukocyte rolling via L-selectin. J. Leukoc. Biol..

[71] Pudelko A., Wisowski G., Olczyk K., Kozma E.M. (2019). The dual role of the glycosaminoglycan chondroitin-6-sulfate in the development, progression and metastasis of cancer. FEBS J..

[72] Widagdo J., Anggono V. (2018). The m6A-epitranscriptomic signature in neurobiology: From neurodevelopment to brain plasticity. J. Neurochem..

[73] Shafik A.M., Zhang F., Guo Z., Dai Q., Pajdzik K., Li Y., Kang Y., Yao B., Wu H., He C. (2021). N6-methyladenosine dynamics in neurodevelopment and aging, and its potential role in Alzheimer’s disease. Genome Biol..

[74] Fawcett J.W., Oohashi T., Pizzorusso T. (2019). The roles of perineuronal nets and the perinodal extracellular matrix in neuronal function. Nat. Rev. Neurosci..

[75] Dzyubenko E., Gottschling C., Faissner A. (2016). Neuron-Glia Interactions in Neural Plasticity: Contributions of Neural Extracellular Matrix and Perineuronal Nets. Neural Plast..

[76] Song I., Dityatev A. (2018). Crosstalk between glia, extracellular matrix and neurons. Brain Res. Bull..

[77] Bonneh-Barkay D., Wiley C.A. (2009). Brain Extracellular Matrix in Neurodegeneration. Brain Pathol..

[78] Clarke L.E., Liddelow S.A., Chakraborty C., Munch A.E., Heiman M., Barres B.A. (2018). Normal aging induces A1-like astrocyte reactivity. Proc. Natl. Acad. Sci. USA.

[79] Bok E., Jo M., Lee S., Lee B.R., Kim J., Kim H.J. (2019). Dietary Restriction and Neuroinflammation: A Potential Mechanistic Link. Int. J. Mol. Sci..

[80] Cahoy J.D., Emery B., Kaushal A., Foo L.C., Zamanian J.L., Christopherson K.S., Xing Y., Lubischer J.L., Krieg P.A., Krupenko S.A. (2008). A transcriptome database for astrocytes, neurons, and oligodendrocytes: A new resource for understanding brain development and function. J. Neurosci..

[81] Sun W., Cornwell A., Li J., Peng S., Osorio M.J., Aalling N., Wang S., Benraiss A., Lou N., Goldman S.A. (2017). SOX9 Is an Astrocyte-Specific Nuclear Marker in the Adult Brain Outside the Neurogenic Regions. J. Neurosci..

[82] Pan H., Xue W., Zhao W., Schachner M. (2020). Expression and function of chondroitin 4-sulfate and chondroitin 6-sulfate in human glioma. FASEB J..

[83] Kai Y., Tomoda K., Yoneyama H., Yoshikawa M., Kimura H. (2015). RNA interference targeting carbohydrate sulfotransferase 3 diminishes macrophage accumulation, inhibits MMP-9 expression and promotes lung recovery in murine pulmonary emphysema. Respir. Res..

