# Supplementary material for: Aging-Associated Changes in Cognition, Expression and Epigenetic Regulation of Chondroitin 6-Sulfotransferase Chst3
Source: Cells. 2022 Jun 27;11(13):2033. doi: 10.3390/cells11132033 (PMC9266018; doi:10.3390/cells11132033)
Supplement: Supplementary file 1 [file cells-11-02033-s001.zip › cells-1730456-supplementary.pdf]

Article

Aging-Associated Changes in Cognition, Expression and Epigenetic Regulation of Chondroitin 6-Sulfotransferase *Chst3*

David Baidoe-Ansah [1], Sadman Sakib [2], Shaobo Jia [1], Hadi Mirzapourdelavar [1], Luisa Strackeljan [1], Andre Fischer [2,3,4], Stepan Aleshin [1,*], Rahul Kaushik [1,5,*] and Alexander Dityatev [1,5,6]

[1] Molecular Neuroplasticity, German Center for Neurodegenerative Diseases (DZNE), 39120 Magdeburg, Germany; david.baidoe-ansah@dzne.de (D.B.-A.); jiashaobo3000@gmail.com (S.J.); hadi.mirzapourdelavar@dzne.de (H.M.); luisastrackeljan@aol.com (L.S.)

[2] Department for Epigenetics and Systems Medicine in Neurodegenerative Diseases, German Center for Neurodegenerative Diseases, 37075 Goettingen, Germany; a.fischer@eni-g.de (A.F.); m.sadman.sakib@gmail.com (S.S.)

[3] Clinic for Psychiatry and Psychotherapy, University Medical Center Goettingen (UMG), 37075 Goettingen, Germany

[4] Cluster of Excellence MBExC, University of Göttingen, 37075 Göttingen, Germany

[5] Center for Behavioral Brain Sciences (CBBS), 39106 Magdeburg, Germany

[6] Medical Faculty, Otto-von-Guericke University, 39120 Magdeburg, Germany

* Correspondence: stepan.aleshin@dzne.de (S.A.); rahul.kaushik.phd@gmail.com (R.K.)

Supplementary Tables:

Table S1. Primary antibodies and their dilutions.

Table S2. Secondary antibodies and their dilutions.

Supplementary Figures:

Figure S1. Downregulation of C6 sulfotransferase *Chst3* in the hippocampus and cortex of 22-24 M old mice;

Figure S2. Impaired long-term NOLT memory in aged mice (22-24 M old mice);

Figure S3. Age-specific correlations in gene expression;

Figure S4. Within and between age gene expression correlation patterns;

Figure S5. Validation of CHST3 antibody specificity;

Figure S6. Comparison of age-related dynamics of *CHST3* expression in different regions of the human brain;

Figure S7. Comparison of age-related dynamics of *Chst3* expression in different murine tissue

Table S1. Primary antibodies and their dilutions.

Antibody/Reagent	Dilution	Species	Provider and Product Number
Versican (VCAN)	1:200	Mouse	The Developmental Studies Hybridoma Bank, 12C5
Parvalbumin	1:500	Chicken	Synaptic System; 195006
vGLUT1	1:1000	Guinea pig	Synaptic Systems; 135304
CHST3	1:200	Rabbit	Proteintech; 18242-1-AP
C6S	1:200	Mouse	Seikagaku; 270423
GFAP	1:500	Chicken	Millipore; AB5541
IBA1	1:500	Guinea pig	Synaptic Systems; 234004
NeuN	1:500	Mouse	Chemicon; MAB377
AnkG	1:500	Guinea pig	Synaptic systems; 386004

Table S2. Secondary antibodies and their dilutions.

Antibody (Alexa Fluor)	Dilution	Species	Provider and Product Number
Anti-Rabbit	1:1000	Goat	Invitrogen; A-11035
Anti-Guinea pig	1:1000	Goat	Invitrogen; A11073, A21105
Anti-Mouse	1:1000	Goat	Invitrogen; A21236, A-21044
Anti-Chicken	1:1000	Goat	Invitrogen; A-21449; Abcam; ab175675

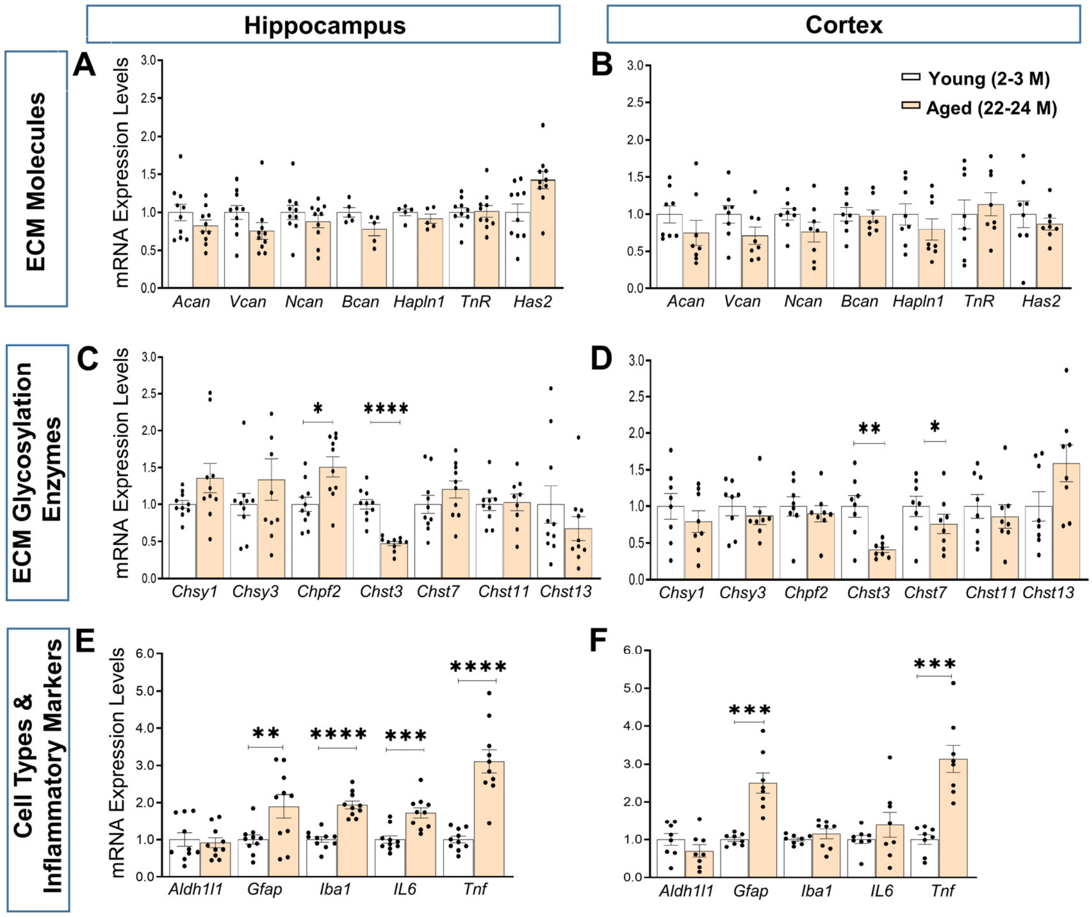

Figure S1. Downregulation of C6 sulfotransferase *Chst3* in the hippocampus and cortex of 22-24 M old mice. The expression of genes involved in the biosynthesis of ECM-related molecules in 22-24 M old mice was investigated using RT-qPCR and normalized to individual GAPDH values. **(A, B)** Genes for core proteins of CSPGs - *Acan, Vcan, Ncan,* and *Bcan* - as well as glycoproteins *Hapln1*, *TnR,* and hyaluronan synthase *Has2* were not significantly different in both the hippocampus and cortex. **(C)** Genes involved in CSPGs GAG chain polymerization were slightly upregulated in the hippocampus, whereas C6 sulfotransferase *Chst3* was downregulated in the hippocampus of 22-24 M old mice. **(D)** However, in the cortex, polymerization complex enzymes were not significantly different, but C6 sulfotransferase *Chst3* was downregulated in 22-24 M old mice compared to 2-3 M old mice. **(E, F)** Inflammatory cytokines and markers for glial activation were upregulated in both the hippocampus and cortex of 22-24 M old mice, except for the cortical expression of *Iba1* and *IL6*. Bar graphs show mean ± SEM values. *$p < 0.05$, **$p < 0.01$, ***$p < 0.001$ and **** $p < 0.0001$ represent significant differences between 2-3 M old mice (n=10 and 8) and 22-24 M old mice (n=10 and 8) for hippocampus and cortex, respectively.

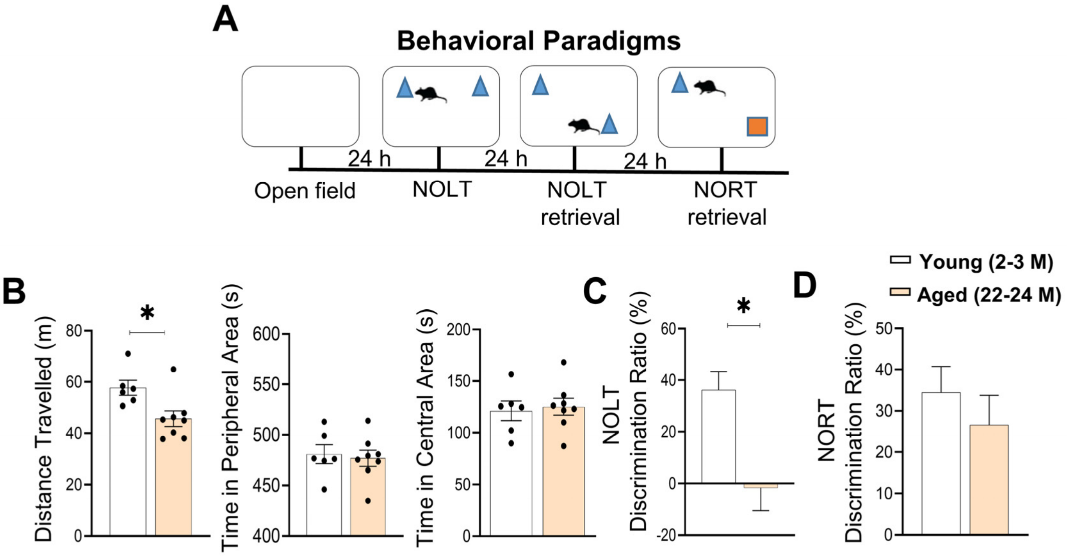

Figure S2. Impaired long-term NOLT memory in aged mice (22-24 M old mice). Long-term memory was tested in 22-24 M old mice and 2-3 M old mice using the novel object location task (NOLT) and novel object recognition task (NORT). **(A)** Time-line for all cognitive tests. **(B)** 22-24 M old mice traveled shorter distances compared to 2-3 M old mice in the open field. **(C)** Moreover, from discrimination ratio analysis, 22-24 M old mice also failed to discriminate between objects at familiar (F) and novel (N) locations during NOLT test. **(D)** However, no difference in discrimination for NORT test occurred between 22-24 M old mice and 2-3 M old mice. Bar graphs show mean ± SEM values for each animal. *$p < 0.05$ represent significant differences between 2-3 M old mice (n=6) and 22-24 M old mice (n=8).

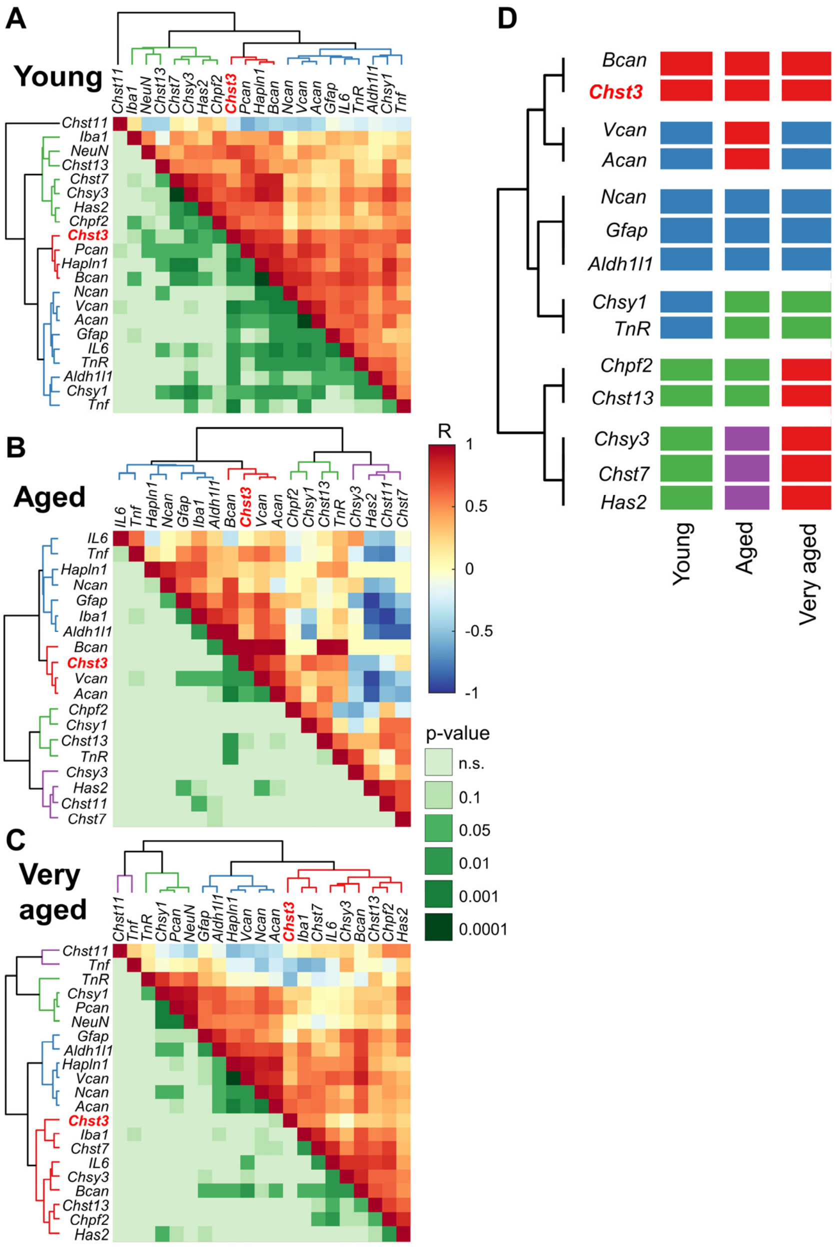

Figure S3. Age-specific correlations in gene expression. (A, B&C) For each age-only gene correlation matrix is shown, the values above and below the diagonal represent the coefficient of the correlation and its statistical significance, respectively. Four clusters were selected for color identification for consistency with the primary age-only clustering. **(D)** Schematic summary of co-clustering results for panels A, B and C. To facilitate discussion and create a general picture, only genes that showed stable co-clustering in different age cohorts were selected from the generic gene pool. The columns denote different ages; the dendrogram groups genes with an identical pattern of co-clustering.

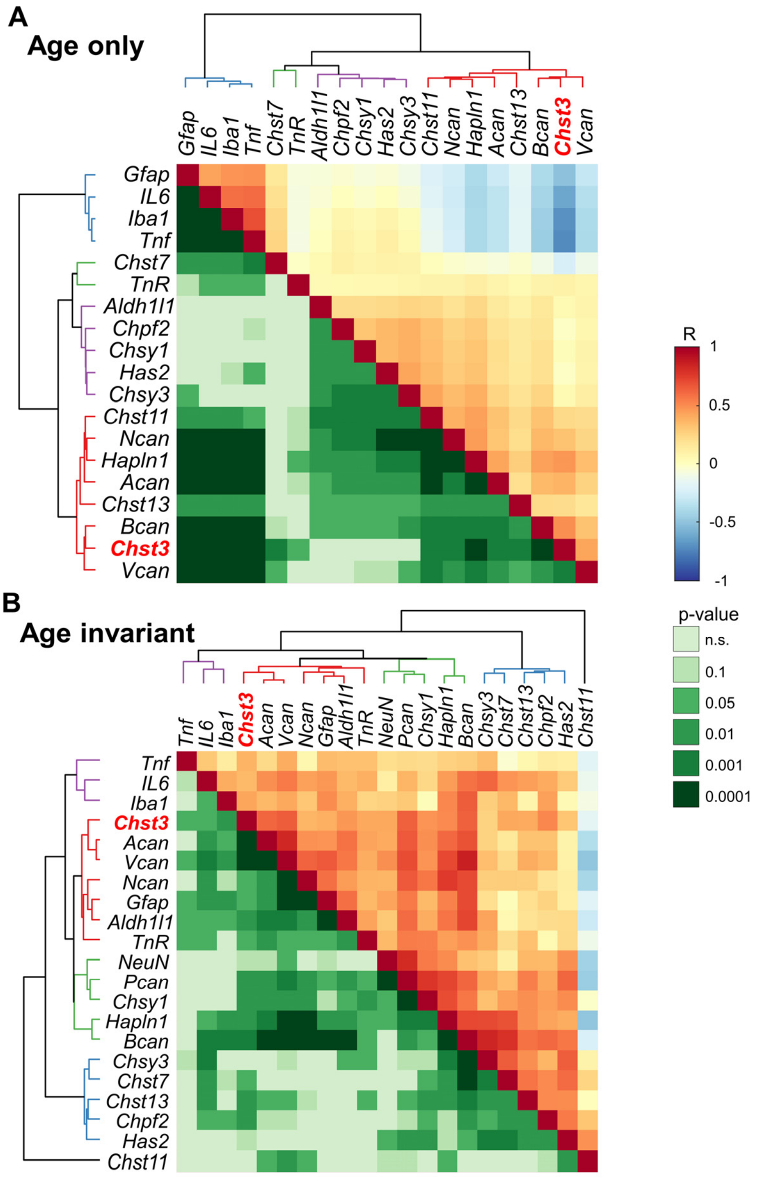

Figure S4. Within and between age gene expression correlation patterns. (A) Age-only heatmap shows the pairwise correlations of genes after removing interactions within the age group (between-age correlations). To compensate for the effects of within-group correlations, gene expression levels were randomly shuffled several times (10^4). The average correlation value is shown in the upper triangle of the heatmap. To obtain the statistical significance shown in the lower triangle of the heatmap, age values for each gene were randomized in the original data. For each of the resulting datasets (10^4), the procedure for calculating age-only correlations described above was repeated. The final 10^4 matrices were used as the H0 distribution to calculate the p-values. **(B)** Age-invariant heatmap shows the pairwise correlations of genes adjusted for exclusion between age groups (within-age correlations). To level out the effect of between-group correlations, gene expression levels were normalized within each group independently, thus eliminating the effect of age on the average level of gene expression. Pairwise correlations were calculated for the resulting datasets, as shown in the upper triangle of the heatmap. To obtain the statistical significance shown in the lower triangle of the heatmap, the approach described above (shuffling) was used.

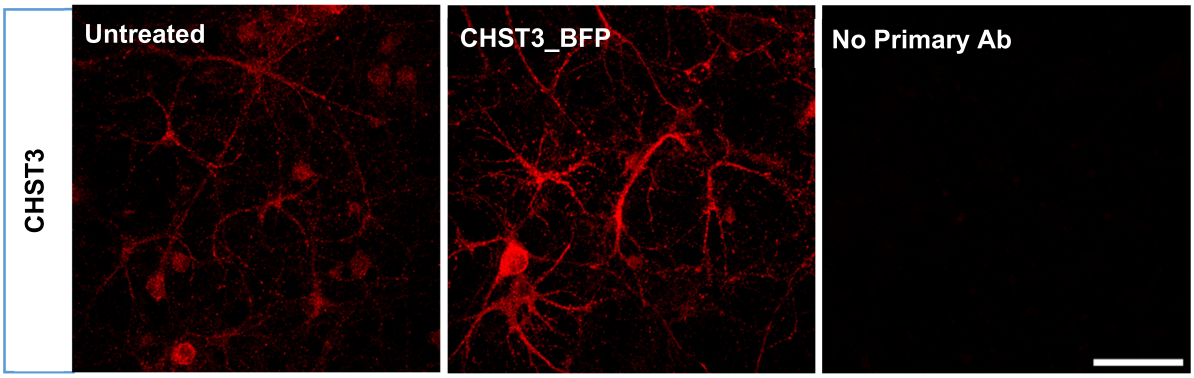

Figure S5. Validation of CHST3 antibody specificity. The CHST3 immunolabeling is increased in neurons and astrocytes overexpressing CHST3 (middle panel) as compared to untreated cells (Scale bar 50 µm).

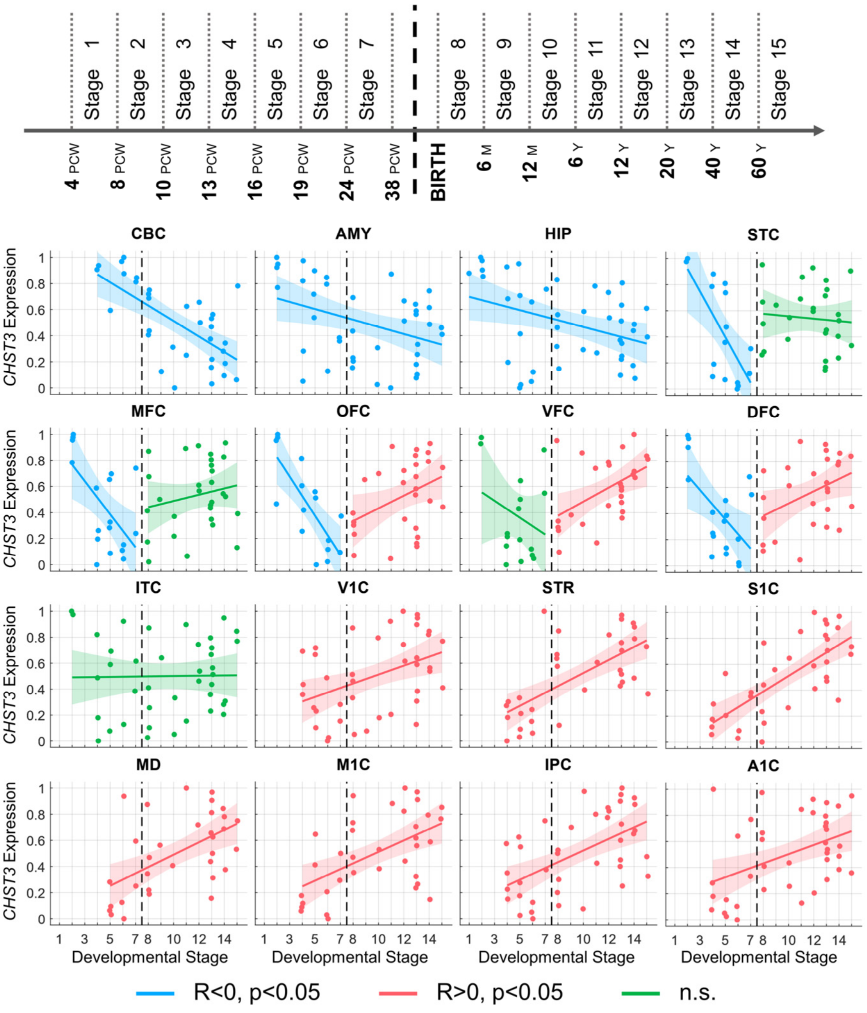

**A1C**	Primary auditory (A1) cortex	**MD**	Mediodorsal nucleus of the thalamus
**AMY**	Amygdala	**MFC**	Medial prefrontal cortex
**CBC**	Cerebellar cortex	**OFC**	Orbital prefrontal cortex
**DFC**	Dorsolateral prefrontal cortex	**S1C**	Primary somatosensory (S1) cortex
**HIP**	Hippocampus	**STC**	Superior temporal cortex
**IPC**	Posterior inferior parietal cortex	**STR**	Striatum
**ITC**	Inferior temporal cortex	**V1C**	Primary visual (V1) cortex
**M1C**	Primary motor (M1) cortex	**VFC**	Ventrolateral prefrontal cortex

Figure S6. Comparison of age-related dynamics of *CHST3* expression in different regions of the human brain. To study the spatiotemporal dynamics of *CHST3* expression in the human brain, data from a paper (Kang et.al. 2011, https://www.nature.com/articles/nature10523) were used to show gene expression during 15 periods covering the periods from embryonic development to late adulthood (top panel), M - postnatal months; PCW - postconceptual weeks; Y - postnatal years. Rank normalized *CHST3* expression values were subjected to regression analysis for pre- and postnatal periods both together and separately. The results of the best models are presented in the middle panel (blue - significant negative correlations, red - significant positive correlations, green - insignificant correlation). Descriptions of the brain region names are given in the table in the bottom panel.

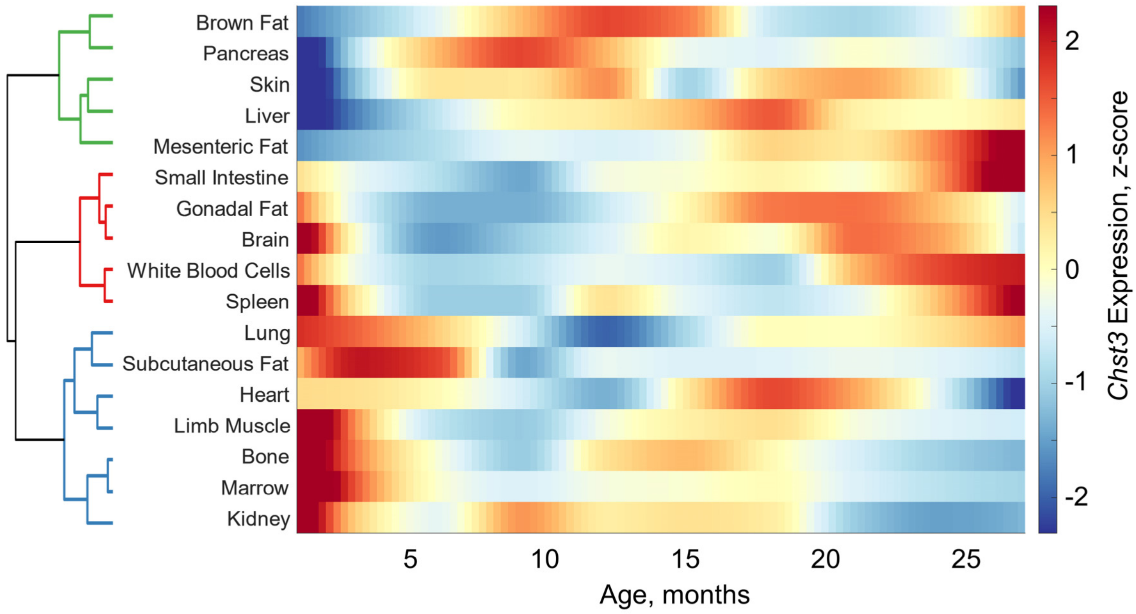

Figure S7. Comparison of age-related dynamics of *Chst3* expression in different mice tissues. To analyze the age-related dynamics of *Chst3* expression in various tissues and organs of mice, we used single-cell transcriptomics data obtained by Schaum et al. (https://www.ncbi.nlm.nih.gov/pmc/articles/PMC7757734/). To estimate *Chst3* trajectories during aging, similar to the original paper, normalized counts from DEseq2 were z-scored and LOESS (Locally weighted scatterplot smoothing) regression was fitted for each trajectory using the median expression per age group in each tissue. The distance matrix between *Chst3* trajectories was computed using the Euclidian distance and hierarchical clustering was performed using the complete method.
